# Genome-wide association study of seedling stage salinity tolerance in *temperate japonica* rice germplasm

**DOI:** 10.1186/s12863-017-0590-7

**Published:** 2018-01-03

**Authors:** Dariga Batayeva, Benedick Labaco, Changrong Ye, Xiaolin Li, Bakdaulet Usenbekov, Aiman Rysbekova, Gulzhamal Dyuskalieva, Georgina Vergara, Russell Reinke, Hei Leung

**Affiliations:** 1 Kazakh State Women’s Teacher Training University, Almaty, 050040 Kazakhstan; 2 International Rice Research Institute, Laguna, 4031 Philippines; 3 Institute of Food Crops, Yunnan Academy of Agricultural Sciences, Kunming, 650205 China; 4 Institute of Plant Biology and Biotechnology, Ministry of Education and Science, Almaty, 050010 Kazakhstan

**Keywords:** Genome-wide association studies, Seedling stage, Salinity tolerance, Candidate gene, *Temperate japonica*

## Abstract

**Background:**

Salinity has a significant impact on rice production in coastal, arid and semi-arid areas in many countries, including countries growing temperate rice, such as Kazakhstan. Recently, the complete genomes of 3000 rice accessions were sequenced through the 3 K rice genome project, and this set included 203 *temperate japonica* rice accessions. To identify salinity-tolerant germplasm and related genes for developing new salinity-tolerant breeding lines for the *temperate japonica* rice growing regions, we evaluated the seedling stage salinity tolerance of these sequenced *temperate japonica* rice accessions, and conducted genome-wide association studies (GWAS) for a series of salinity tolerance related traits.

**Results:**

There were 27 accessions performed well (SES < 5.0) under moderate salinity stress (EC12), and 5 accessions were tolerant under both EC12 and EC18. A total of 26 QTLs were identified for 9 measured traits. Eleven of these QTLs were co-located with known salinity tolerance genes. QTL/gene clusters were observed on chromosome 1, 2, 3, 6, 8 and 9. Six candidate genes were identified for five promising QTLs. The alleles of major QTL *Saltol* and gene *O*_*S*_*HKT1;5* (*SKC1*) for Na^+^/K^+^ ratio identified in *indica* rice accessions were different from those in the *temperate japonica* rice accessions used in this study.

**Conclusion:**

Salinity tolerant *temperate japonica* rice accessions were identified in this study, these accessions are important resources for breeding programs. SNPs located in the promising QTLs and candidate genes could be used for future gene validation and marker assisted selection. This study provided useful information for future studies on genetics and breeding of salinity tolerance in *temperate japonica* rice.

**Electronic supplementary material:**

The online version of this article (10.1186/s12863-017-0590-7) contains supplementary material, which is available to authorized users.

## Background

Rice is grown worldwide in areas where temperatures are suitable and irrigation water is available. This includes many areas with soil salinity problems. Rice is rated as a salt-sensitive crop, especially at early seedling and reproductive stages [1, 2]. The threshold for salt stress in rice is 3 dS/m, with a 12% reduction in yield for each unit increase in dS/m beyond this value [3]. The narrow genetic pool of *japonica* germplasm for salinity tolerance and the extent of the salinity challenge are common to many rice-growing regions. Rice production is limited by soil salinity problems in many tropical countries such as Bangladesh and India, and temperate countries such as Korea, Japan, Iran and Kazakhstan [4]. For example, the rice growing regions in Kazakhstan are located in the most northern regions of rice cultivation on earth (from Kyzyl-Orda 44°48’N to Balkhash 46°00’N) with total area of about 100,000 ha and paddy production of 300,000–500,000 tons per year. Soil salinity and low temperatures at the early growing stage are the main environmental problems for rice production in Kazakhstan [5]. However, most of the previous studies on rice salinity tolerance have been conducted in tropical regions using *indica* accessions. Some salinity-tolerant rice accessions (Nona Bokra, Pokkali, PSBRc50, FL478) have been identified and used in breeding programs, and many salinity-tolerant *indica* rice varieties have been developed. IRRI released a significant number of salt-tolerant varieties from 2011 to 2013. These include three varieties released in Bangladesh (Bina dhan 10, BRRI dhan 55 and 61), one variety in India (Luna Sankhi), eight ‘Salinas’ varieties in the Philippines and one variety in Gambia [6]. However, very few salinity-tolerant *japonica* rice accessions have been identified, such as Harra (Spain), Agami (Egypt) and Daeyabyeo (Korea) [7]. Thus, it is important to identify salinity-tolerant *japonica* rice germplasm and use them to develop salinity-tolerant *japonica* varieties for temperate rice growing regions.

Rice is very sensitive to salinity at the seedling stage. Soil salinity usually causes stunting or even death of seedlings [8]. Based on the ability of seedlings to grow in salinized nutrient solution, a screening protocol was developed and widely used for germplasm screening, breeding selection and genetic studies [9]. The typical mechanism of salinity tolerance in rice is the Na^+^ exclusion or uptake reduction, and increased absorption of K^+^ to maintain a suitable Na^+^-K^+^ balance in the shoot [10]. Thus, shoot Na^+^, K^+^ content and Na^+^/K^+^ ratio are also used as valid criterion in measuring salinity tolerance in rice [9].

Salinity tolerance is a complex trait controlled by quantitative trait loci (QTLs). Many QTLs for salinity tolerance have been identified [11–24]. However, most of the QTLs were identified in bi-parental populations derived from *indica* accessions such as Pokkali and Nona Bokra, very few QTLs for salinity tolerance have been identified in *temperate japonica* accessions [15, 25]. A recent study showed that significant differences in phenotypic response to salinity exist within the *japonica* accessions of European Rice Core collection (ERCC) [26]. More genetic studies should be carried out to understand the salinity tolerance QTLs in *temperate japonica* accessions and to facilitate the subsequent use of these QTLs in breeding programs.

Recent development of high density markers such as SNPs enables identification of trait-marker association through association mapping, and GWAS for many important agronomic traits have been reported [27–29]. With the collaboration among the Chinese Academy of Agricultural Sciences (CAAS), the Beijing Genomics Institute (BGI) and the International Rice Research Institute (IRRI), 3000 rice accessions of known diversity were systematically sequenced [30]. After alignment with the reference genome (Nipponbare), around 18.9 million SNPs were identified. This database provided valuable information for understanding the genetic mechanism of useful traits in these diversity accessions.

The objectives of this study were to screen a set of *temperate japonica* accessions which had been fully sequenced, to identify SNPs associated with seedling stage salinity tolerance, and to identify potential candidate genes for the promising QTLs. We selected the subset of *temperate japonica* accessions from the 3000 sequenced accessions, evaluated the seedling stage salinity tolerance of these accessions using 16 different traits (indexes), and conducted genome-wide association studies between these traits and core SNP markers.

## Methods

### Plant materials

The 3 K Rice Genomes Project sequenced 3000 rice genomes with an average sequencing depth of 14×. The dataset includes publicly available genome sequences derived from 3000 accessions of rice with global representation (from 89 countries) of genetic and functional diversity [30]. Phylogenetic analyses based on SNP data confirmed differentiation of the *O. sativa* gene pool into 5 varietal groups – *indica*, *aus/boro*, *basmati/sadri*, *tropical japonica* and *temperate japonica* [31]. There were 203 accessions in the *temperate japonica* group (Additional file 1: Figure S1). We requested all the sequenced *temperate japonica* accessions, however, seed availability was limited to 191 accessions, and these accessions were used in this study (Additional file 2: Table S1).

### Evaluation of salinity tolerance at seedling stage

The seeds of selected accessions were treated at 50 °C for 5 days in an oven to break seed dormancy. Two pre-germinated seeds were sown in each well on a styrofoam seedling float that was placed on a tray filled with distilled water. Each accession was sown into four wells, and each seeding float contained 20 accessions plus one tolerant check variety (FL478) and one susceptible check variety (NSIC Rc222). The experiment was conducted in the phytotron glasshouse maintained at 29/21 **°C** day/night temperature and minimum relative humidity of 70%. The experimental design was a randomized complete block design (RCBD) with 2 treatments (control and salt treatment), and 3 replications for each treatment. For the salt treatment, four days after seeding (DAS), we replaced the distilled water with salinized Peter’s Professional™ nutrient solution (20 N-20P-20 K) as hydroponic solution at a rate of 1 g per liter water and with 400 mg of ferrous sulfate, the electrical conductivity was adjusted to EC = 6 dSm^−1^ by adding NaCl to the nutrient solution, and the pH was adjusted to 5.1 daily. At 7 DAS, the hydroponic solution was changed to maintain at EC = 12 dSm^−1^. After two weeks in EC 12 dSm^−1^, the visual reactions of plant to salinity stress were evaluated using percentage of damage (PD) and the Standard Evaluation Score (SES) for salinity tolerance (Additional file 3: Table S2) [32]. After scoring, one seedling of each replicate was sampled for measurement of shoot Na^+^ (SNa) and K^+^ (SK) content. Then the electrical conductivity of the hydroponic solution was changed to maintain at EC = 18 dSm^−1^. A final scoring of PD and SES was done after 7 days exposure to EC = 18 dSm^−1^. The control was always maintained in distilled water (EC0), and all samplings and measurements were the same as salt treatment.

### Measurement of Na^+^ and K^+^ content

One seedling from each replicate was sampled and washed 3 times with distilled water. The seedling was put in paper bags and dried in an oven at 70 °C for at least 3 days. Dry weight of the seedling (SDW) was measured, and then ten milligrams of dried leaf were cut into small crumbs (<1 cm) and placed in 10 ml Falcon tubes, 10 ml 0.1 N acetic acid (CH_3_COOH) was added to each tube, then heated in a waterbath at 90 °C for 2 h. The solution was filtered through Whatman No1 filter paper placed in a glass funnel, and the filtration was collected in a 10 ml flask as stock solution. The stock solution was then diluted 10 times (1 ml of stock solution in 9 ml of nanopure water), then sodium and potassium content were measured using a flame spectrophotometer (Sherwood Model 420).

### Genome sequence data

Approximately 18.9 million single nucleotide polymorphisms (SNPs) were discovered in 3000 rice genomes when aligned to the reference genome of the *temperate japonica* accession Nipponbare [31]. The SNPs and allele information were organized into a SNP-Seek system [33, 34]. The core SNP V2.1 of the selected 191 *temperate japonica* rice accessions were downloaded from the SNP-seek system and used for data analysis.

### Data analysis

For the phenotypic data of salinity tolerance related traits, the mean value of three replications was calculated and used for genome-wide association study. The relative reduction rate of each trait was calculated as (control-treatment)×100/control. Basic statistical information of the traits is shown in Additional file 4: Table S3. The correlations among different traits were calculated by using MINITAB V14.0 (Minitab Inc.).

The core SNP dataset of the selected 191 *temperate japonica* accessions included 365,710 SNPs. There were a large proportion of missing calls along with many heterozygous SNPs detected in the dataset. Fifteen accessions with high heterozygotes (>1%) and high missing sites (>25%) were removed. The Trait Analysis by Association Evolution and Linkage (TASSEL) program version 5.2.18 [35], was used to filter the sites at a maximum count of 158 of the 176 remaining accessions, which accounts for sites in which 90% of the accessions have a call and a minimum frequency of 0.05 for the minor allele. The above criteria resulted in 68,786 filtered sites. Finally 176 accessions with 68,786 SNPs were used for making cladogram tree with neighbor-joining and generating kinship matrix with centered IBS (default) [36]. Principal components analysis was done using these filtered 68,786 SNPs with default settings. A united data file with the genotype and phenotype of the accessions was created by using union join. The united file along with kinship matrix was used to analyze marker-trait associations using a mixed linear model (MLM) [37]. The compression level was set to optimum level, and variance component estimation was set to P3D. The significant threshold was set at *p* < 0.0001 (−log10 *p*-value >4.0) and/or SNP marker R^2^ ≥ 0.1 [38]. The QQ plot is shown in Additional file 5: Figure S2. The identified QTLs were named using the CGSNL nomenclature [39], and mapped on the rice genome using Mapchart 2.30 [40]. A promising QTL was considered when many SNPs were lined up near the peak of the QTL. The chromosomes and QTL regions of the promising QTLs were selected and re-analyzed using general linear model (GLM) with 1000 times permutation. Candidate genes (near peak SNPs) for the promising QTLs were searched on the rice genome browser in the rice SNP seek database [34] and literature reports.

## Results

### Diversity and population structure of selected *temperate japonica* rice accessions

**Fig. 1:**
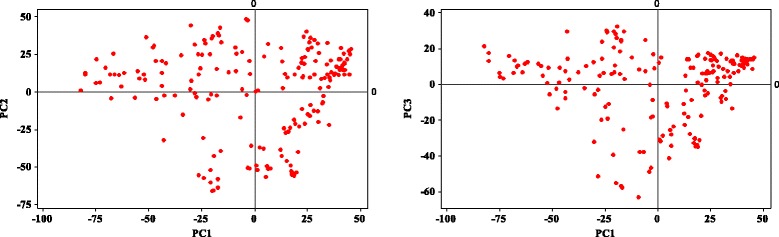
Principal component analysis (PCA) of 176 temperate japonica rice varieties using 68,786 SNPs

There were 203 *temperate japonica* accessions included in the 3000 sequenced genomes. However, the seeds of 12 accessions were not available at the time of request, and 15 accessions showed high heterozygous SNP sites and a high rate of missing data. Thus, 176 *temperate japonica* accessions from 32 countries were used for analysis. The principal component analysis of the 176 accessions showed that all the accessions were closely linked in one group, and no significant sub-group was classified (Fig. 1). When the origin of the accessions were considered, accessions from most of the countries were randomly distributed, except that some of the Chinese and Korean accessions were closely linked (Additional file 6: Figure S3). Thus, population structure was not considered in the following GWAS analysis.

### Salinity tolerance of selected *temperate japonica* rice accessions

There was a wide range of variation in salinity tolerance at seedling stage among the 176 *temperate japonica* accessions. However, based on the visual evaluation, only 3 of the evaluated accessions (Nep Ngau from Vietnam, Bai Mang Ai Zhong from China and Shinchiku Iku 97 from Taiwan) were as tolerant as the tolerant check variety FL478 at EC12 (SES = 3), and none of them was as tolerant as FL478 at EC18 (Table 1). Twenty-seven salinity tolerant accessions were listed in Additional file 7: Table S4. Among them, only 7 accessions were tolerant (SES ≤ 5) at both EC12 and EC18 including Bai Mang Ai Zhong and Shinchiku Iku 97.

**Table 1:** Number of accessions in different SES categories

SES^a^	3	3–5	5	5–7	7	7–9	9	Total
EC12	3	18	20	57	27	31	20	176
EC18	0	5	7	14	20	35	95	176

^a^The SES of tolerant check variety was 3, while SES of susceptible check variety was 9 at both EC12 and EC18

Among the evaluated traits, shoot dry weight under normal conditions (control) was not correlated with other traits under salt treatment except shoot dry weight (Table 2). Since SES scores and percentage of damage (PD) are based on visual evaluation of the injury of the seedlings, SES was correlated with PD. Shoot Na^+^ and K^+^ content under normal conditions (control) were not significantly correlated with PD and SES score, however leaf Na^+^ content and Na^+^/K^+^ ratio were correlated with PD and SES under salt stress. SES12 and SES18 are significantly correlated, scoring once at EC12 treatment is enough for evaluating the salinity tolerance of a variety. Shoot dry weight (include its relative deduction compared to control) and shoot Na^+^ content at EC12 treatment are significantly correlated with PD and SES, thus SDW12, SDWD and SNa12 are good indices for evaluating salinity tolerance of rice varieties. For EC12 treatment, shoot dry weight (SDW12) and its relative deduction (SDWD) are significantly correlated with shoot Na^+^ (SNa12), K^+^ (SK12) content and Na^+^/K^+^ ratio (SNa/K12), the change of Na^+^, K^+^ content and their ratio in the shoot directly affected the growth of the plant.

**Table 2:** Pearson correlation among salinity tolerance related traits

	SES12	SES18	PD12	PD18	SDW0	SDW12	SDWD	SNa0	SNa12	SNaD	SK0	SK12	SKD	SNa/K0	SNa/K12
SES18^a^	0.582***^b^														
PD12	0.967***	0.566***													
PD18	0.582***	0.995***	0.563***												
SDW0	0.072	0.097	0.074	0.101											
SDW12	−0.467***	−0.323***	−0.466***	−0.328***	0.236**										
SDWD	0.332***	0.274***	0.336***	0.280***	0.657***	−0.476***									
SNa0	0.121	0.081	0.130	0.089	0.076	−0.032	0.135								
SNa12	0.362***	0.229**	0.334***	0.241**	0.013	−0.394***	0.296***	0.124							
SNaD	−0.174*	−0.135	−0.141	−0.141	0.052	0.343***	−0.178*	0.517***	−0.677***						
SK0	0.078	0.039	0.068	0.043	0.040	−0.026	0.073	0.128	0.124	−0.004					
SK12	−0.159*	−0.097	−0.175*	−0.099	−0.033	0.315***	−0.245**	0.138	−0.059	0.135	0.088				
SKD	0.114	0.024	0.121	0.025	0.031	−0.104	0.108	0.065	0.182*	−0.120	0.597***	−0.266***			
SNa/K0	0.043	0.082	0.042	0.082	0.066	−0.022	0.073	0.372***	−0.098	0.325***	−0.611***	−0.031	−0.698***		
SNa/K12	0.186*	0.156*	0.176*	0.169*	−0.017	−0.386***	0.239**	−0.068	0.614***	−0.500***	0.020	−0.603***	0.282***	−0.102	
SNa/KD	−0.083	−0.080	−0.121	−0.089	−0.004	0.256**	−0.174*	0.254**	−0.426***	0.560***	−0.336***	0.456***	−0.359***	0.411***	−0.692***

^a^SES = standard evaluation score, PD = percentage of damage, SDW = shoot dry weight, SNa = shoot Na^+^ content, SK = shoot K^+^ content. Numbers 0, 12 and 18 are treatments of EC0 (control), EC12 and EC18. SDWD, SNaD, SKD and Na^+^/K^+^D are relative deduction of the related traits at EC12 compared to control

^b^*significant at *p* < 0.05, **significant at *P* < 0.01, ***significant at *P* < 0.001

### Genome-wide association study of traits related to seedling stage salinity tolerance

#### Percentage of damage and standard evaluation score

For the percentage of damage of the seedlings after salinity treatment at EC12 for 2 weeks, no significant QTLs was identified. However, three QTLs on chromosomes 2, 4 and 11 were identified for percentage of damage of the seedlings after salinity treatment at EC18 (Table 3, Additional file 8: Figure S4).

**Table 3:** Identified QTLs for the assessed salinity responsive traits

Trait	QTL	Chr	QTL interval (Mb)	Peak position (bp)	SNP Marker	F value	p value	Marker R^2^
PD18	*qPD18_2.1*	2	24.38–24.51	24,382,186	10,224,382,186	18.11	3.53E-05	0.1119
PD18	*qPD18_4.1*	4	17.08–17.09	17,083,910	10,417,083,910	19.43	1.87E-05	0.1134
PD18	*qPD18_11.1*	11	24.64–24.65	24,645,753	11,124,645,753	19.92	1.50E-05	0.1174
SES18	*qSES18_2.1*	2	24.38–24.51	24,382,186	10,224,382,186	20.23	1.31E-05	0.1255
SES18	*qSES18_4.1*	4	17.06–17.09	17,083,910	10,417,083,910	19.28	2.01E-05	0.1127
SES18	*qSES18_11.1*	11	24.64–24.65	24,645,753	11,124,645,753	20.89	9.55E-06	0.1234
SDW0	*qSDW0_2.1*	2	6.98–7.12	6,992,556	10,206,992,556	16.02	9.29E-05	0.092
SDW0	*qSDW0_9.1*	9	7.77–7.78	7,779,630	10,907,779,630	8.57	2.95E-04	0.1111
SNa^+^0	*qSNa0_2.1*	2	4.79–6.56	5,610,544	10,205,610,544	22.43	4.54E-06	0.1283
SNa^+^0	*qSNa0_6.1*	6	27.17–29.49	27,397,942	10,627,397,942	19.86	1.52E-05	0.12
SNa^+^0	*qSNa0_8.1*	8	21.49–21.51	21,498,898	10,821,498,898	16.21	8.59E-05	0.0997
SNa^+^0	*qSNa0_8.2*	8	26.56–26.57	26,568,663	10,826,568,663	10.12	7.13E-05	0.1225
SNa^+^0	*qSNa0_9.1*	9	6.77–7.42	7,411,725	10,907,411,725	16.83	6.47E-05	0.1043
SNa^+^12	*qSNa12_4.1*	4	20.33–20.57	20,374,274	10,420,374,274	11.62	1.91E-05	0.1427
SNa^+^12	*qSNa12_10.1*	10	14.91–16.19	15,893,435	11,015,893,435	16.29	8.30E-05	0.0988
SNa^+^D	*qSNaD_5.1*	5	10.28–11.25	10,287,595	10,510,287,595	17.9	3.83E-05	0.1062
SNa^+^D	*qSNaD_6.1*	6	23.74–23.85	23,820,480	10,623,820,480	16.49	7.53E-05	0.0984
SK^+^D	*qSKD_1.1*	1	32.96–38.59	38,584,909	10,138,584,909	20.12	1.33E-05	0.1155
SK^+^D	*qSKD_2.1*	2	24.49–25.43	24,686,915	10,224,686,915	30.49	1.23E-07	0.1754
SK^+^D	*qSKD_2.2*	2	28.40–28.92	28,694,339	10,228,694,339	24.25	1.97E-06	0.1391
SK^+^D	*qSKD_3.1*	3	3.69–3.81	3,805,384	10,303,805,384	28.01	3.69E-07	0.1611
SK^+^D	*qSKD_4.1*	4	4.49–4.72	4,509,563	10,404,509,563	30.72	4.08E-12	0.3525
SK^+^D	*qSKD_12.1*	12	16.55–17.62	16,556,527	11,216,556,527	18.41	2.95E-05	0.1056
SNa^+^/K^+^0	*qSNa/K0_3.1*	3	3.72–3.81	3,805,384	10,303,805,384	29.01	2.37E-07	0.168
SNa^+^/K^+^0	*qSNa/K0_11.1*	11	17.62–17.63	17,624,102	11,117,624,102	8.4	3.45E-04	0.1012
SNa^+^/K^+^D	*qSNa/KD_3.1*	3	23.88–24.08	24,079,731	10,324,079,731	15.67	1.10E-04	0.1019

Same as PD, no QTL was identified for SES of EC12 treatment, but three QTLs were identified on chromosomes 2, 4 and 11 for EC18 treatment. Since the SES and PD were significantly correlated, QTLs identified for SES and PD were also located in the same regions on the chromosomes.

#### Shoot dry weight

There was a wide range of variation of shoot dry weight among the accessions. Under normal conditions (control), two QTLs related to shoot dry weight were identified on chromosomes 2 and 9. However, no QTL was identified for shoot dry weight after salinity treatment and the relative reduction of shoot dry weight.

#### Shoot Na^+^ and K^+^ content and Na^+^/K^+^ ratio

QTLs for shoot Na^+^ content were identified on chromosomes 2, 6, 8 and 9 under normal conditions (control) and on chromosomes 4 and 10 under salinity treatment (EC12). After salinity treatment, the leaf Na^+^ content increased comparing to control, and QTLs for this increment were identified on chromosomes 5 and 6.

For shoot K^+^ content, no QTL was identified for control and salinity treatment. After salinity treatment, the leaf K^+^ content increased, and QTLs for this increment were identified on chromosomes 1, 2, 3, 4 and 12.

QTLs for shoot Na^+^/K^+^ ratio were identified on chromosomes 3 and 11 under normal conditions (control), but no QTL was identified for salinity treatment (EC12). After salinity treatment, the leaf Na^+^/K^+^ ratio varied among accessions, with some showing higher ratios and some lower. One QTL for the change of Na^+^/K^+^ ratio were identified on chromosomes 3.

Some of the identified QTLs on chromosomes 2 (*qPD18_2.1*, *qSES18_2.1* and *qSKD_2.1*), 3 (*qSNa/K0_3.1* and *qSKD_3.1*), 4 (*qPD18_4.1* and *qSES18_4.1*), 9 (*qSNa0_9.1* and *qSDW0_9.1*) and 11 (*qPD18_11.1* and *qSES18_11.1*) are located in the same position or very closely linked (Table 3, Additional file 9: Figure S5).

#### Candidate genes for promising QTLs

**Fig. 2:**
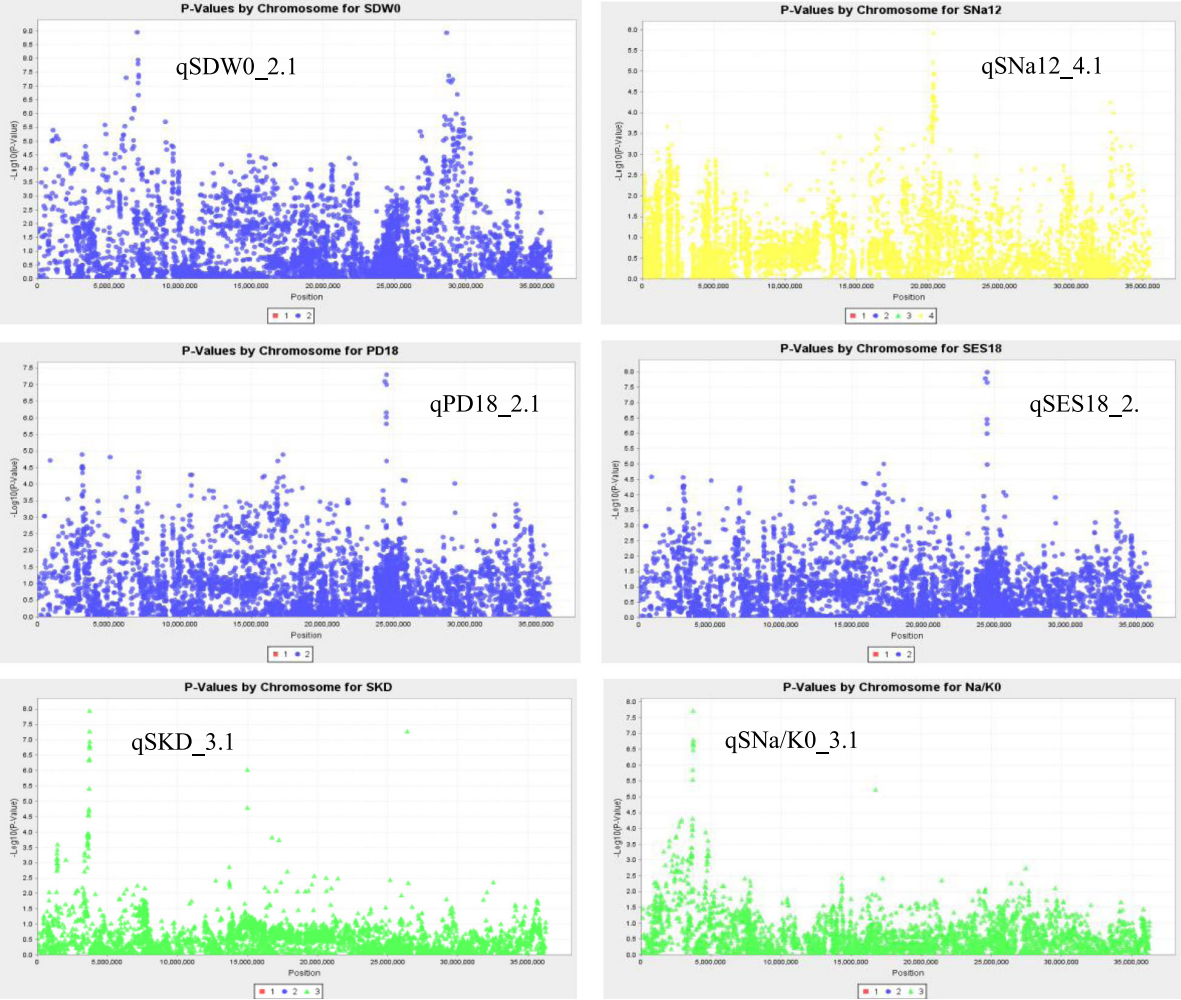
Manhattan plots of promising QTLs. Manhattan plots of selected QTLs were generated by using GLM with 1000 times of permutation

Based on the significant SNPs in the QTL regions, six QTLs on chromosomes 2, 3 and 4 were promising (Fig. 2). By searching previous reported genes related to salinity tolerance, six candidate genes located at or near the peak SNPs were identified for five promising QTLs (Table 4). There are 1–5 SNPs within these genes, these SNP markers could be used for validate the candidate genes.

**Table 4:** Candidate genes for promising QTLs

TLs	Genes in QTL	Putative candidate genes	Gene annotation	SNPs	Tolerant hyplotype
*qPD18_2.1* *qSES18_2.1*	15	*LOC_Os02g4053*	MYB transcription factor	10,224,580,840(A/G)	G
				10,224,580,869(G/T)	G
				10,224,581,422(C/T)	T
				10,224,582,310(C/A)	A
		*LOC_Os02g40730*	Ammonium transporter (*OsAMT1.3*)	10,224,691,455(C/A)	A
				10,224,691,620(C/T)	T
				10,224,691,683(T/G)	G
				10,224,692,377(G/T)	G
				10,224,692,615(G/A)	A
*qSKD_3.1* *qSNa/K0_3.1*	18	*LOC_Os03g07450*	Homeobox associated leucine zipper	10,303,786,888(C/T)	T
		*LOC_Os03g07480*	Sucrose transporter	10,303,802,066(T/G)	G
*qSNa12_4.1*	38	*LOC_Os04g33640*	Glycosyl hydrolases gene family	10,420,374,274(C/T)	T
				10,420,376,435(G/A)	G
		*LOC_Os04g33720*	Glycosyl hydrolases gene family	10,420,414,566(A/G)	G

## Discussions

It is difficult to introduce salinity tolerance of *indica* accessions into *japonica* accessions that have high grain quality and high yield [41]. Inter sub-specific crossing barriers between *indica* and *japonica* background also limit the ready introgression of salinity tolerance QTLs [42]. Breeding methods have been limited by the lack of salinity-tolerant *japonica* accessions. To date, very few salinity-tolerant rice accessions have been identified and used in breeding programs, and of those which have been identified, most are *indica* accessions. In this study, we identified some salinity-tolerant *temperate japonica* rice accessions such as, Shinchiku Iku 97, Lomellino, Norin 21, 81A32 and 36,037–1, showing good tolerance at both EC12 and EC18. These accessions could be important sources of salinity tolerance for future temperate rice breeding programs.

Many salinity tolerance QTLs have been identified in different studies [11–24]. However, most of them were mapped using different populations and markers. It is difficult to compare the positions of those QTLs and the QTLs identified in our current study because different markers were used. Fortunately, many genes related to rice salinity tolerance have been published in the QTL Annotation Rice Online database [43], and those genes can be tracked on the genome browser of rice SNP-seek database [34]. We compared the positions of the QTLs identified in this study and the salinity tolerance related genes in the genome browser. The results showed many QTL/gene clusters on chromosomes 1, 2, 3, 4, 6, 8, 9 and 11. Some of the QTLs identified in this study were co-located with salinity tolerance genes on chromosomes 1, 2, 3, 6, 8 and 9 (Additional file 9: Figure S5). These QTL/gene clusters might be important loci for salinity tolerance in *temperate japonica* rice varies, and related SNP markers could be used for QTL validation and marker assisted selection in breeding programs.

By comparing the SNPs in the QTL regions, six QTLs with a large number of SNPs showing low *p* values were identified as very promising (Fig. 2). Six candidate genes located at or near the peak SNPs were identified for five QTLs (Table 4). In the QTL region of *qSDW0_2.1*, there is no known gene controlling shoot dry weight, only a homeobox domain containing protein gene (*Os02g13310*) was down-regulated under desiccation and salt stress [44], however, no SNP was found in this gene.

In the QTL region of *qPD18_2.1* and *qSES18_2.1*, gene *Os02g40530* is an MYB transcription factor which was up-regulated under drought and/or salt stresses [45]. Another gene *Os02g40730* is a Ammonium transporter (*OsAMT1.3*) which was significantly up-regulated under salt treatment of rice varieties FL478 and IR29, since high Cl^−^ concentrations can inhibit NO_3_^−^ transport, up-regulation of ammonium uptake would be beneficial to withstand salt stress [46].

In the QTL region of *qSKD_3.1* and *qSNa/K0_3.1*, gene *Os03g07450* is a homeobox associated leucine zipper gene, which was down-regulated under desiccation and salt stress [44]. Gene *Os03g07480* encodes a sucrose transporter (SUT). Sucrose allocation between tissues is a fundamental process in all multicellular organisms. Thus, sucrose transporter genes play an essential role in phloem loading and assimilate partitioning [47]. In sweet sorghum, salinity-tolerant species accumulate more sucrose by enhancing the synthesis and reducing the decomposition of sucrose under salt stress, while salt-sensitive species show enhanced decomposition of sucrose [48]. There are nine sucrose transporter genes in the rice genome [49, 50]. Sucrose transporter genes are essential for long distance sucrose transport and for osmo-protectant activities during drought and salinity stresses. Cultivars with higher expression of SUTs should be able to tolerate drought and salinity stresses better than those with lower expression [51].

In the QTL region of *qSNa12_4.1*, genes *Os04g33640* and *Os04g33720* belong to gene family of glycosyl hydrolases (GH). Glycosyl hydrolase functions in both the biosynthetic and hydrolytic pathways of raffinose metabolism, especially under certain abiotic stress conditions such as drought, high salinity or high temperature [52, 53]. Gene *Os04g33640* (Glycosyl hydrolase family 17) showed the highest alternate transcripts among 579 genes in a cold-stressed leaf library [54]. *Os04g33720* was specifically expressed in lowland rice, but not expressed in upland rice [55]. *Os04g33720* was also shown to play an important signalling role in leaves subject to biotic and abiotic stresses [56, 57].

Although previous studies showed that some of the candidate genes were directly or indirectly related to salinity tolerance, the function of many of these genes are still unknown. Further study is needed to validate the expression and effect of these genes under salt stress.

Among the previously reported salinity tolerance QTLs, a major QTL for shoot K^+^ concentration (*qSKC1* or *O*_*S*_*HKT1;5*) explaining 40.1% of the total phenotypic variance was identified on chromosome 1 and subsequently investigated by map-based cloning. It was shown to encode a *HTK1* Na^+^ transporter and is involved in Na^+^ and K^+^ homeostasis [12, 58, 59]. Another QTL controlling shoot Na^+^/K^+^ ratio, *SalTol*, was originally identified using a RIL population derived from the cross IR29/Pokkali, explained 64% of the phenotypic variation [9], and was validated to the same region of *SKC1* [11]. Haq et al. [14] also identified a QTL for Na^+^/K^+^ ratio between 11.1 and 14.6 Mb on chromosome 1 from a *tropical japonica* variety Moroberekan. A recent association mapping effort using varieties from the *japonica* cultivar group has also identified the *SalTol* genomic region as controlling important aspects of salinity tolerance [26]. It has been suggested that *O*_*S*_*HKT1;5* may be the causal gene underlying these QTLs and functionally effective for salt tolerance across both *indica* and *japonica* accessions. However, Platten et al. (2013) identified seven major and three minor alleles of *O*_*S*_*HKT1;5* gene from *Oryza sativa* and *Oryza glaberrima*, the *aromatic* allele conferred the highest leaf Na^+^ exclusion, and the *japonica* allele the least [60]. A salinity-tolerant allele of the *O*_*S*_*HKT1;5* gene was also found in the wild rice species *O. rufipogon and O. nivara* [61]. In our current study, no QTL for Na^+^/K^+^ ratio was identified in the chromosomal region of these QTLs/genes. There are 6 SNPs in the *O*_*S*_*HKT1;5* gene *Oso1g20160*, and 2 of them were polymorphic between the salinity-tolerant *indica* rice varieties in which *qSKC1* and *SalTol* were identified, Nona Bokra and Pokkali. The haplotype of the 176 *temperate japonica* rice accessions were not the same as Nona Bokna and Pokkali, suggesting that the allele of *O*_*S*_*HKT1;5* gene was not functional in the 176 *temperate japonica* rice accessions used in this study (Additional file 10: Table S5). A previous study showed that SNP mutations may cause the destabilization of a transmembrane domain in *O*_*S*_*HKT1;5* and increase the probability of the *O*_*S*_*HKT1;5* phosphorylation [62]. Rice lines containing Leu in position 395 of *O*_*S*_*HKT1;5* gene exhibited higher shoot Na^+^ concentration than those containing Val [63]. The change of Asn to Asp in *HKT1*-type transporters also established altered cation selectivity and uptake dynamics, which enabled some crucifer species to acquire improved salt tolerance [64]. Thus, the SNP haplotype variation in the *O*_*S*_*HKT1;5* alleles may render this gene non-functional in the *temperate japonica* accessions used in this study.

Under moderate salinity stress (EC12), some *temperate japonica* rice accessions achieved the same level of salinity tolerance as the check variety FL478, despite not having the same alleles at the *SKC1* loci associated with Na^+^/K^+^ ratio. This suggests differences between *indica* and *japonica* subspecies in the effect of QTLs and genes involved in salinity tolerance. It is important to identify those *temperate japonica* specific salinity tolerance QTLs/genes that can significantly improve the salinity tolerance of future *temperate japonica* rice breeding lines.

## Conclusions

In this study, we evaluated the seedling stage salinity tolerance of a subset of sequenced *temperate japonica* rice from 3000 sequenced rice accessions, and did genome-wide association study for 16 salinity responsive traits. Twenty-seven salinity-tolerant accessions were identified, and 26 QTLs related to 9 salinity tolerance traits were mapped on chromosomes 1, 2, 3, 4, 6, 8, 9 and 11. Six candidate genes were identified for five promising QTLs. The salinity tolerant varieties and QTLs could be used in related breeding programs. This study provided useful information for future studies on genetics and breeding of salinity tolerance in *temperate japonica* rice.

## Additional files

Additional file 1: **Figure S1.** Selection of 203 temperate japonica from the 3000 sequenced rice accessions. Left is the archaeopteryx tree of 3000 sequenced rice accessions using 365,710 SNPs, and the right is the archaeopteryx tree of 176 *temperate japonica* accessions using 68,786 SNPs. (DOCX 133 kb)

Additional file 2: **Table S1.** List of temperate japonica accessions used. (DOCX 28 kb)

Additional file 3: **Table S2.** Standard evaluation score (SES) of visual salt injury at seedling stage. (DOCX 12 kb)

Additional file 4: **Table S3.** Statistics of the measured traits. (DOCX 14 kb)

Additional file 5: **Figure S2.** QQ plot of the measured traits. (DOCX 57 kb)

Additional file 6: **Figure S3.** Archaeopteryx tree of 176 *temperate japonica* rice accessions using 68,786 SNPs. The origin country of the accession was shown after the sequence ID. (DOCX 697 kb)

Additional file 7: **Table S4.** List of salinity tolerant accessions at seedling stage based on SES12. (DOCX 16 kb)

Additional file 8: **Figure S4.** Manhattan plots of different traits related to salinity tolerance at seedling stage. The genotyping and phenotyping data along with kinship matrix were analyzed using mixed linear model (MLM). (DOCX 1043 kb)

Additional file 9: **Figure S5.** Distribution of identified QTLs on chromosomes. The Os genes are genes related to salinity tolerance from QTARO database (http://qtaro.abr.affrc.go.jp) and rice SNP-seek database (http://snp-seek.irri.org/). The number on the left is the position of the QTL/gene in Mb. (DOCX 711 kb)

Additional file 10: **Table S5.** Hyplotype of SKC1 gene (Oso1g20160, OsHKT1 Na^+^ transporter) in salinity-tolerant varieties (Nona Bokra and Pokkali) and 191 *temperate japonica* accessions. (DOCX 35 kb)

## Abbreviations

GWAS: Genome-wide association study
PD: Percentage of damage
QTL: Quantitative trait locus
QTLs: Quantitative trait loci
SDW: Shoot dry weight
SES: Standard evaluation score
SNP: Single nucleotide polymorphism
