# Supplementary figures and images for: Genome-wide association study of seedling stage salinity tolerance in temperate japonica rice germplasm
Source: BMC Genet. 2018 Jan 3;19:2. doi: 10.1186/s12863-017-0590-7 (PMC5753436; doi:10.1186/s12863-017-0590-7)

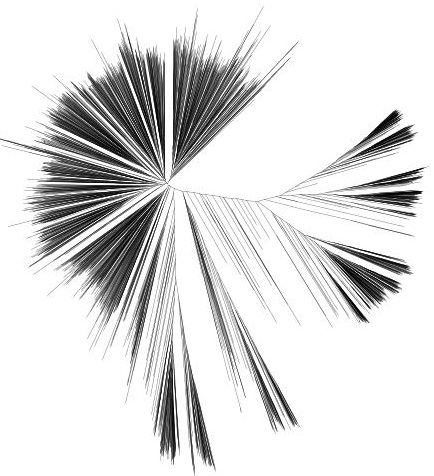

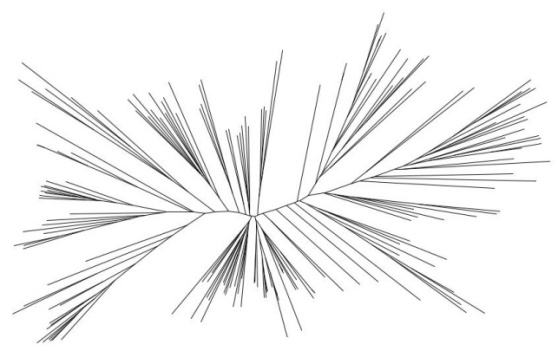


Aus/Boro

Basmati/Sadri

Tropical japonica

Temperate japonica

Indica

Supplement: Supplementary file 1 — Figure S1. Selection of 203 temperate japonica from the 3000 sequenced rice accessions. Left is the archaeopteryx tree of 3000 sequenced rice accessions using 365,710 SNPs, and the right is the archaeopteryx tree of 176 temperate japonica accessions using 68,786 SNPs. (DOCX 133 kb) [file 12863_2017_590_MOESM1_ESM.docx]

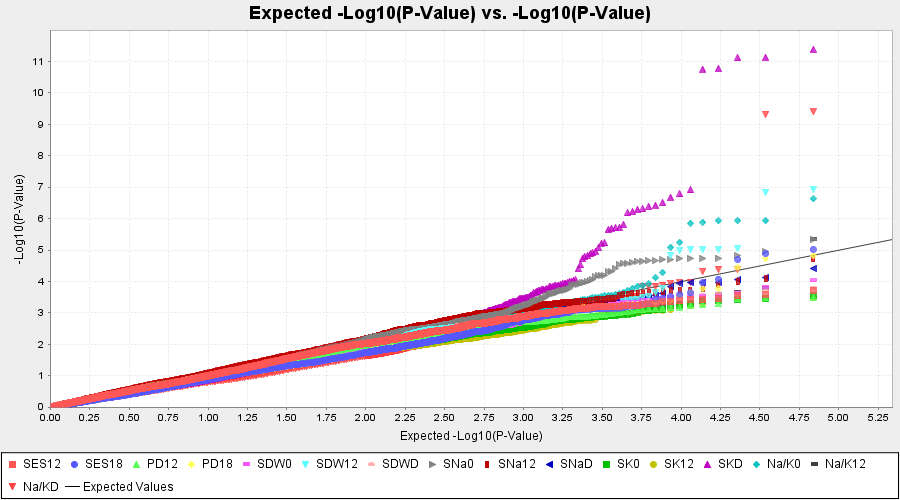

Supplement: Supplementary file 5 — Figure S2. QQ plot of the measured traits. (DOCX 57 kb) [file 12863_2017_590_MOESM5_ESM.docx]

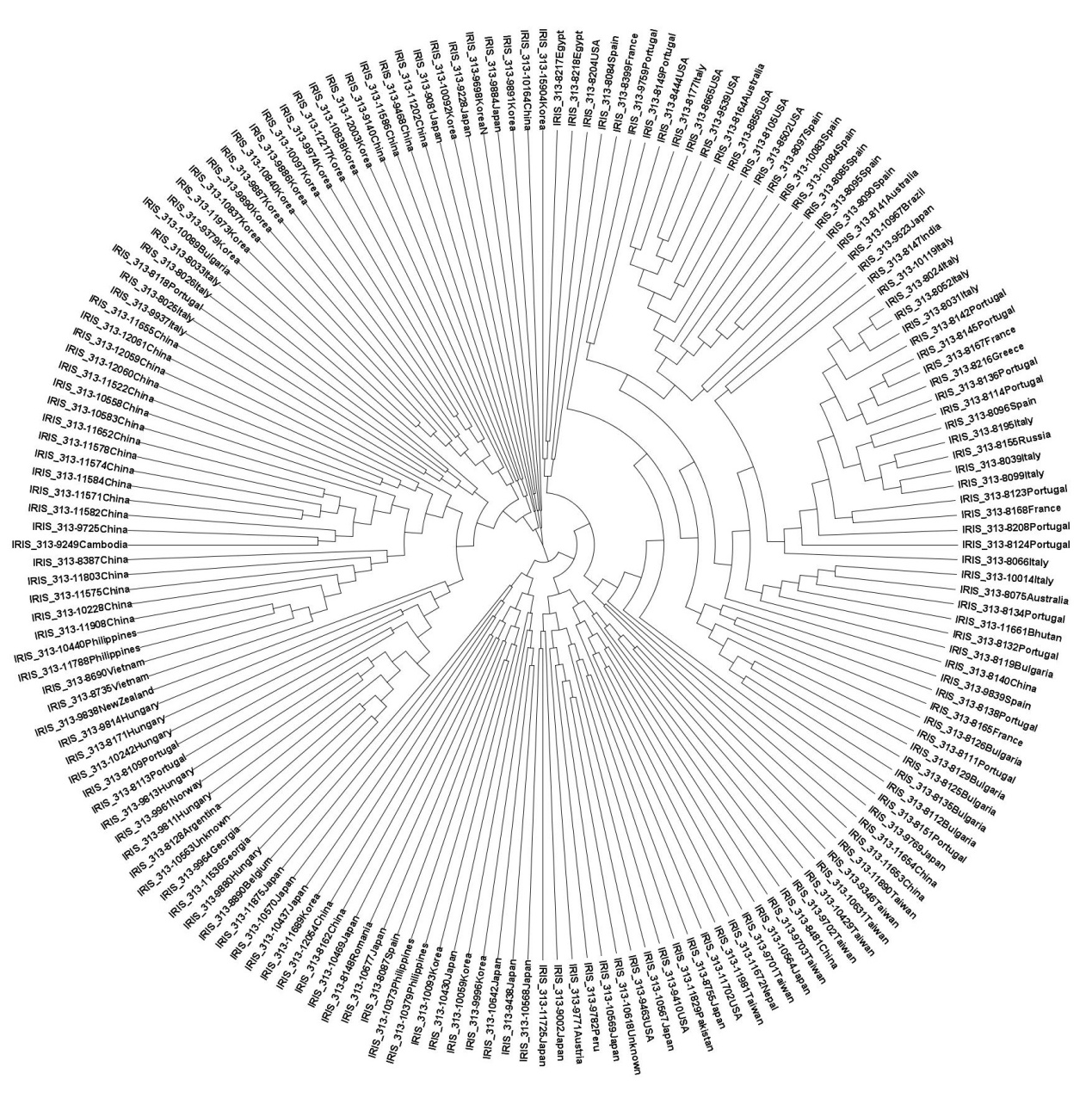

Supplement: Supplementary file 6 — Figure S3. Archaeopteryx tree of 176 temperate japonica rice accessions using 68,786 SNPs. The origin country of the accession was shown after the sequence ID. (DOCX 697 kb) [file 12863_2017_590_MOESM6_ESM.docx]

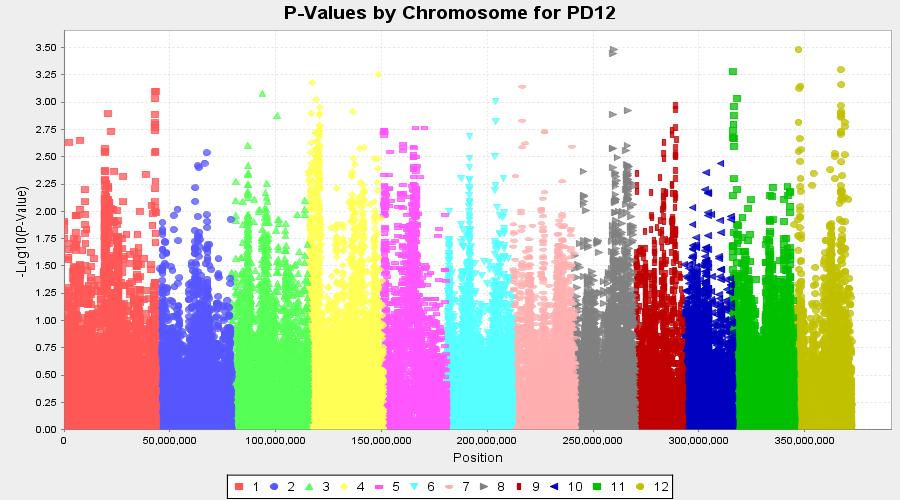

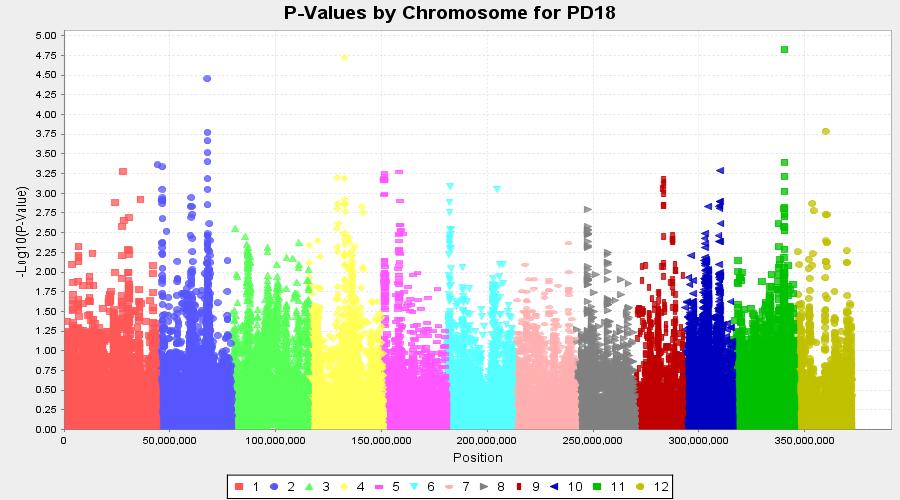


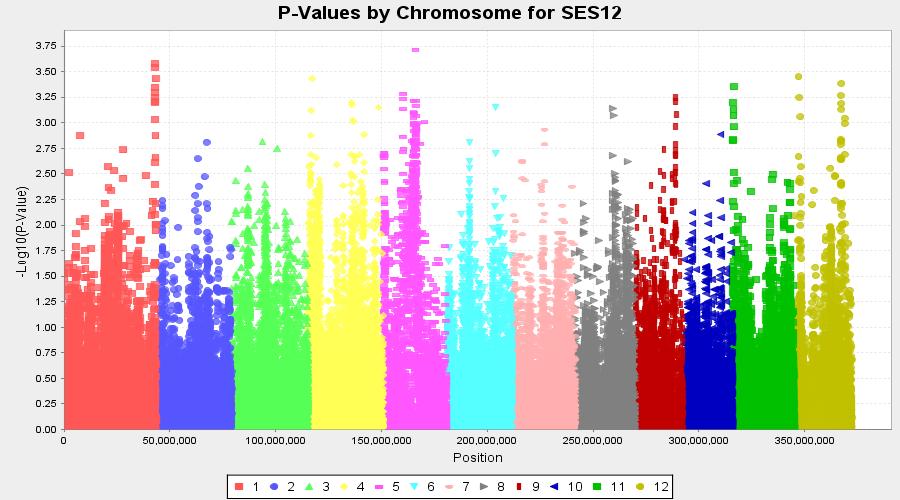


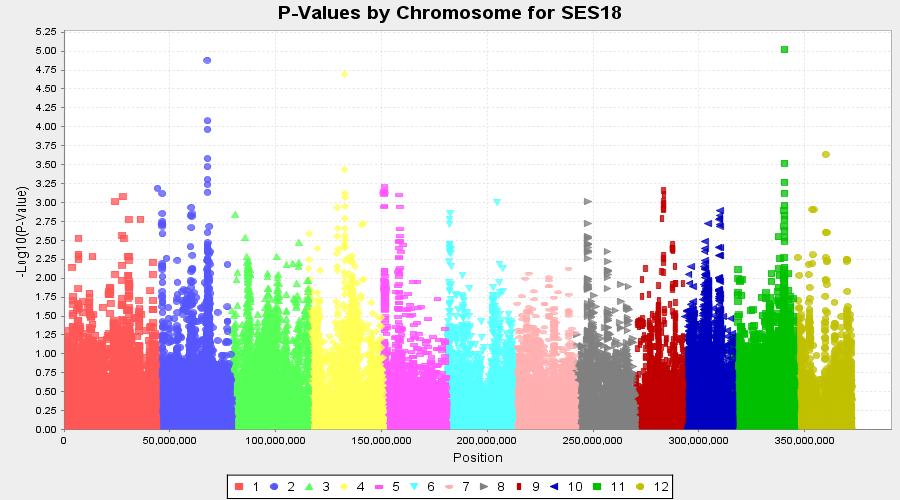


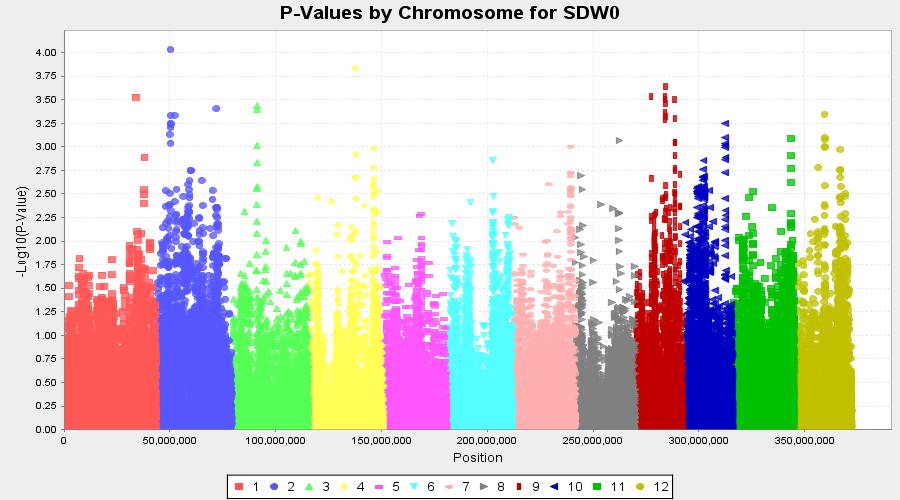

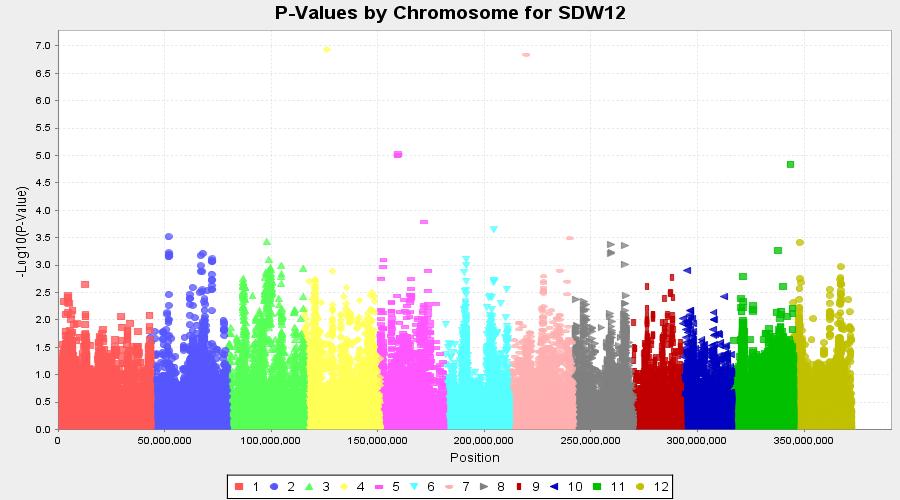

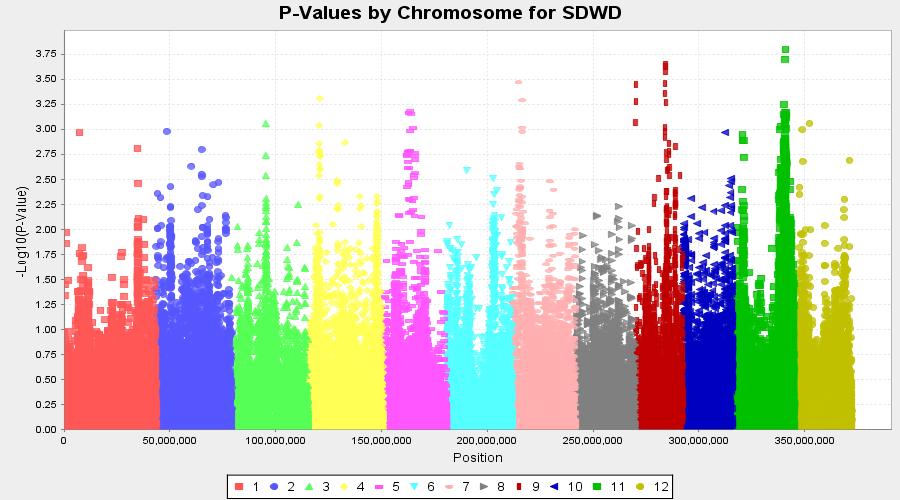

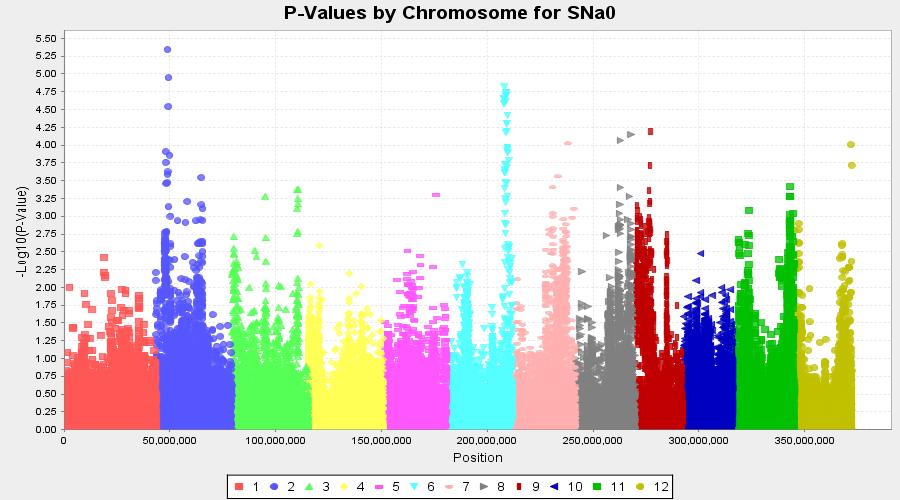

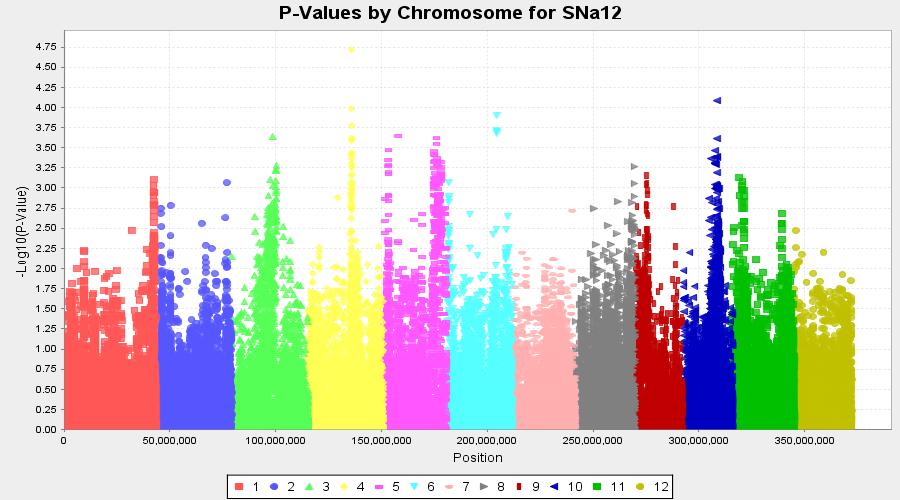

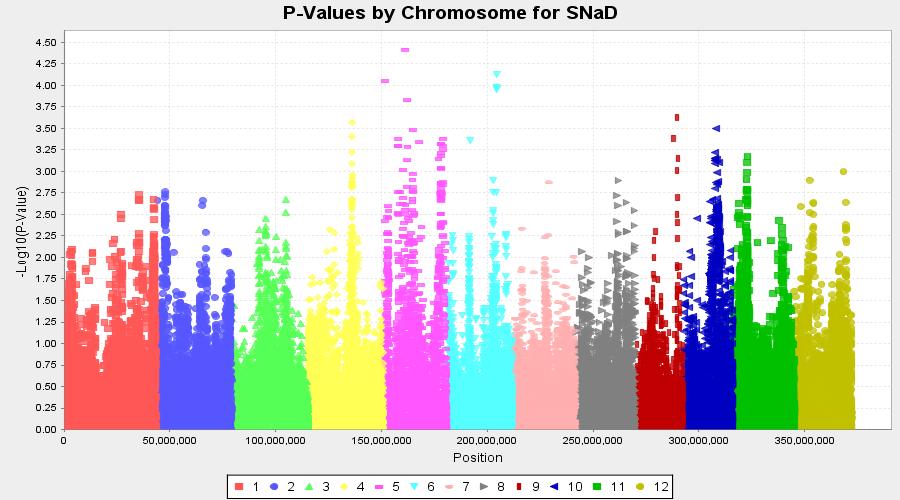

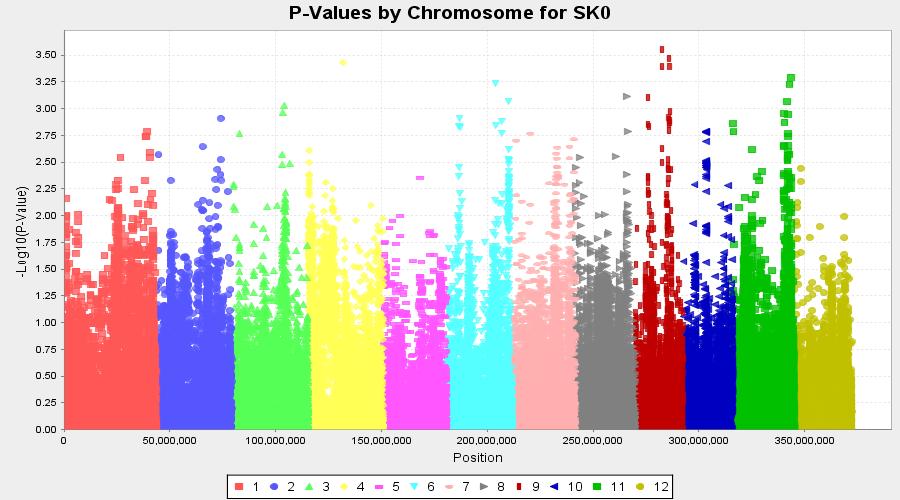

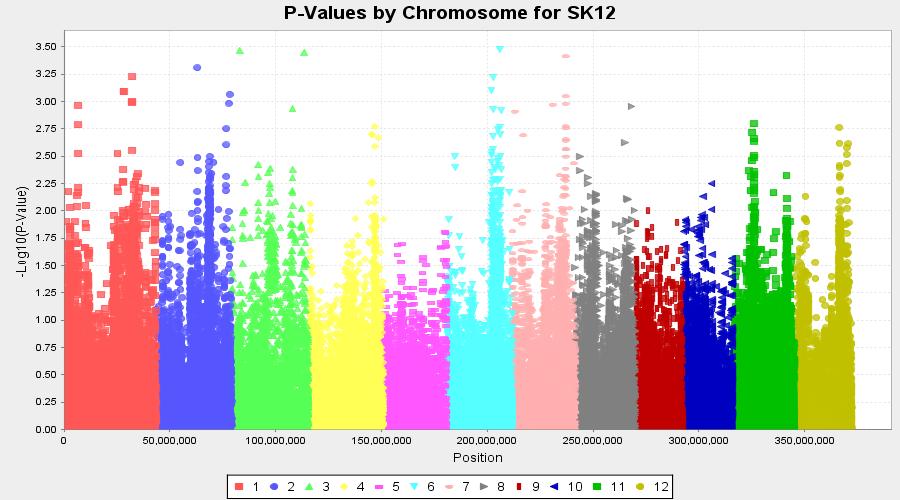

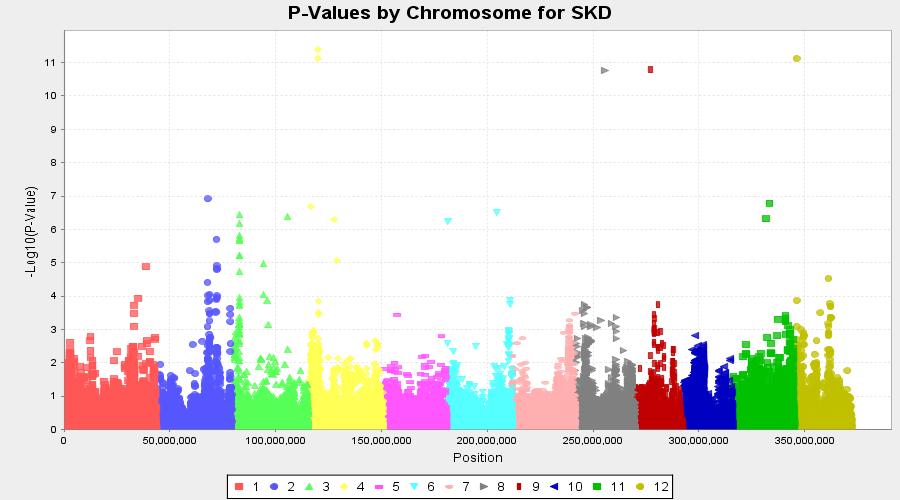


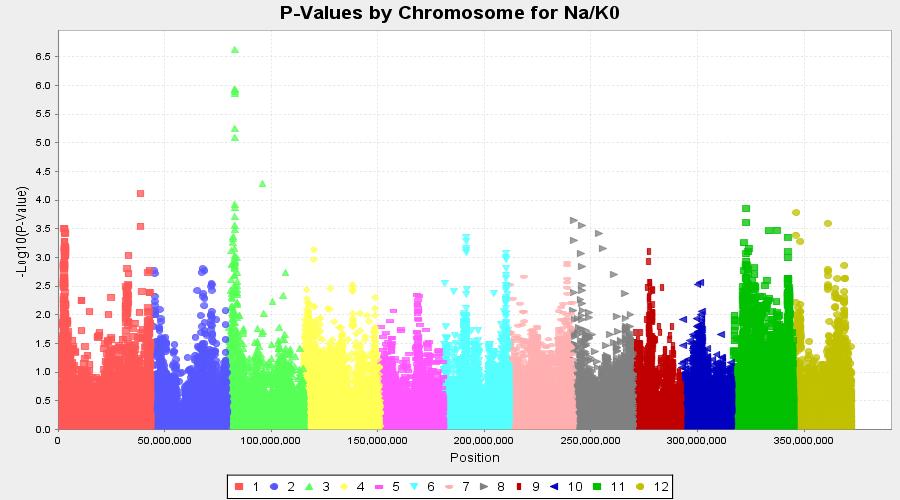


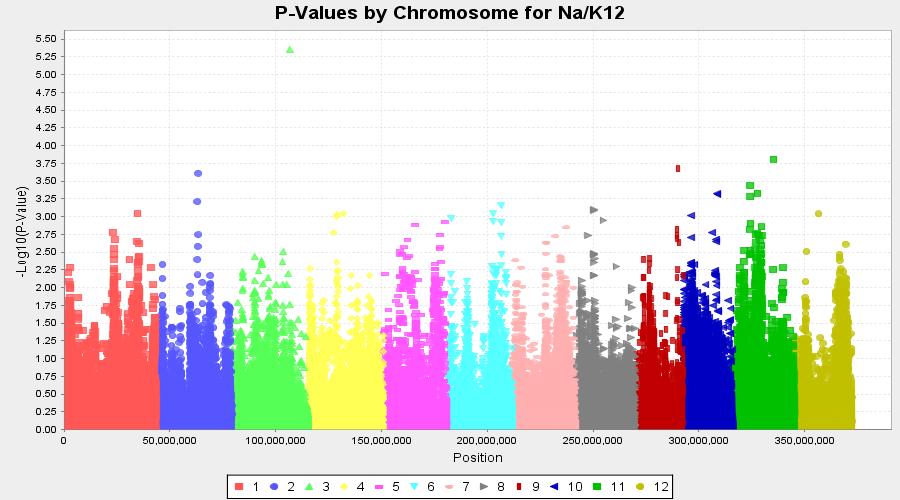


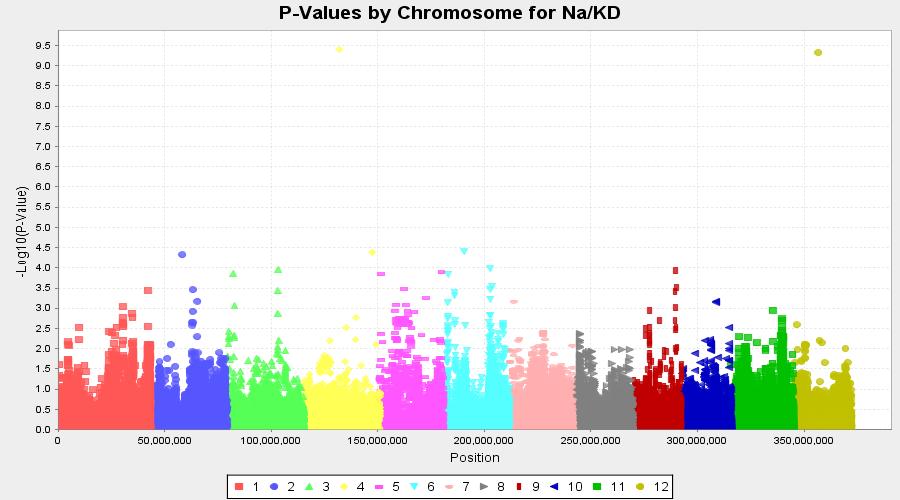

Supplement: Supplementary file 8 — Figure S4. Manhattan plots of different traits related to salinity tolerance at seedling stage. The genotyping and phenotyping data along with kinship matrix were analyzed using mixed linear model (MLM). (DOCX 1043 kb) [file 12863_2017_590_MOESM8_ESM.docx]

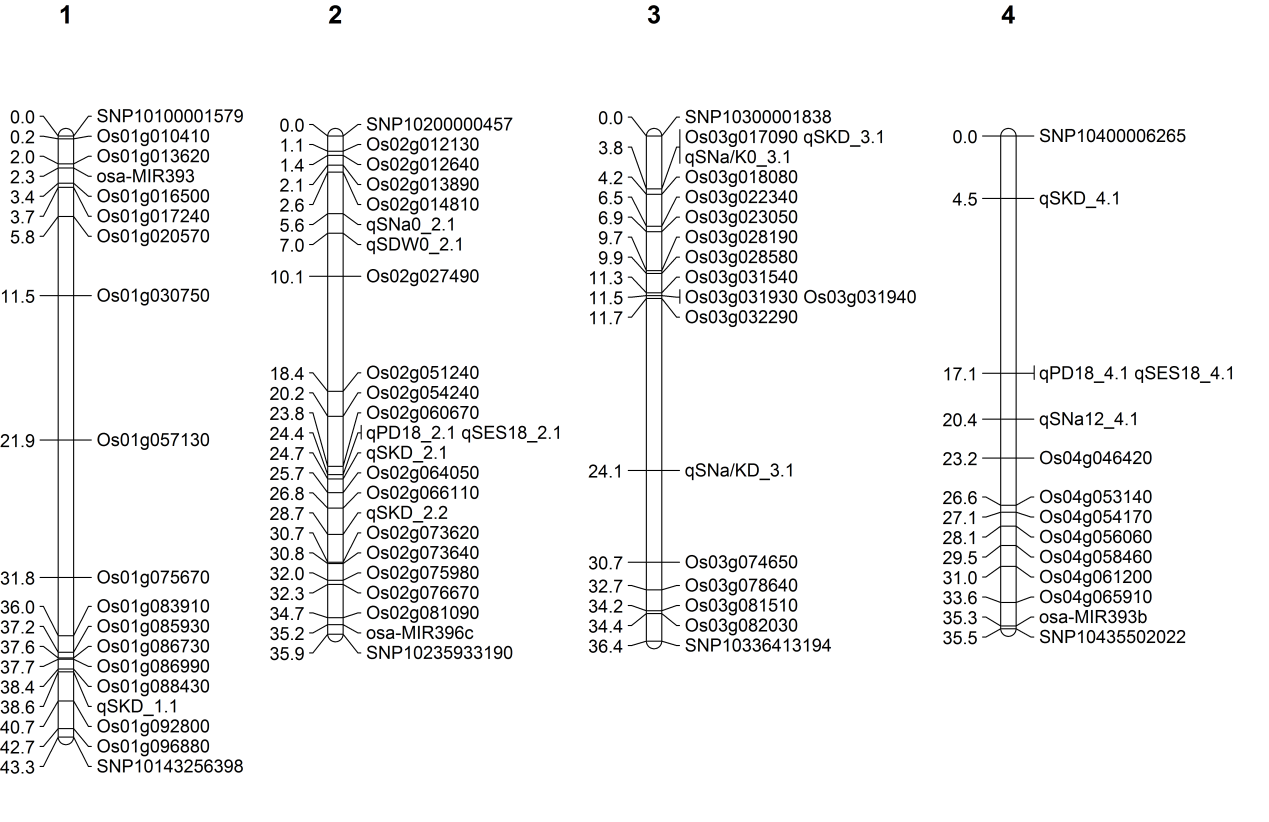


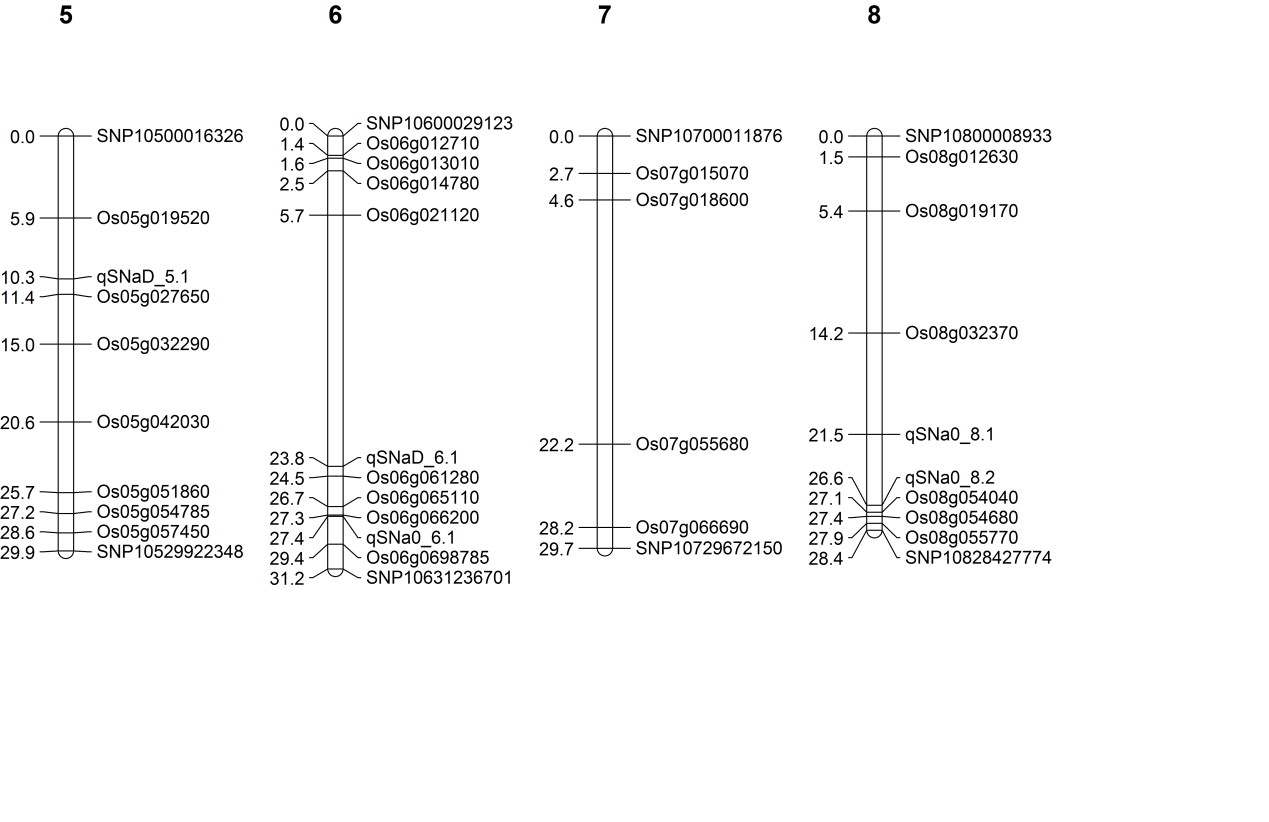


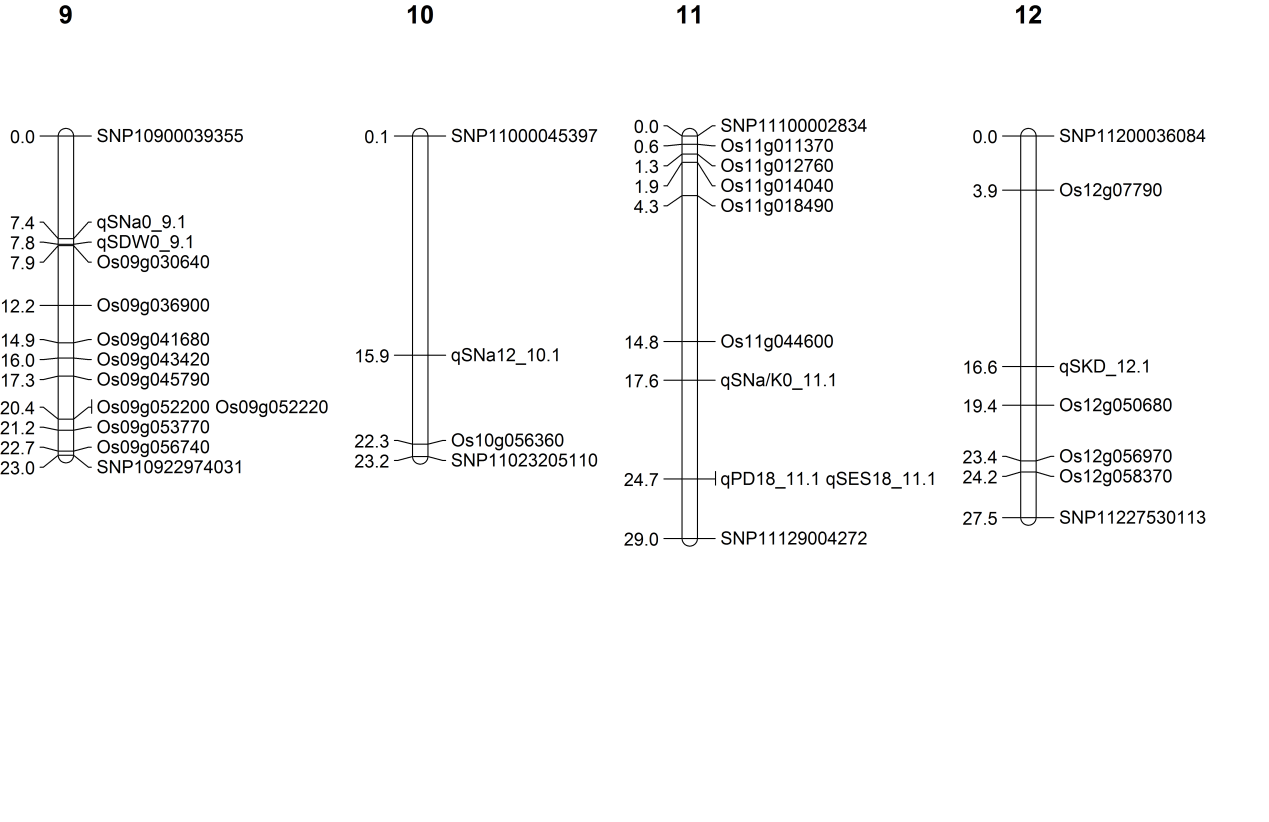

Supplement: Supplementary file 9 — Figure S5. Distribution of identified QTLs on chromosomes. The Os genes are genes related to salinity tolerance from QTARO database (http://qtaro.abr.affrc.go.jp) and rice SNP-seek database (http://snp-seek.irri.org/). The number on the left is the position of the QTL/gene in Mb. (DOCX 711 kb) [file 12863_2017_590_MOESM9_ESM.docx]
