# Supplementary material for: Genome-wide association study of seedling stage salinity tolerance in temperate japonica rice germplasm
Source: BMC Genet. 2018 Jan 3;19:2. doi: 10.1186/s12863-017-0590-7 (PMC5753436; doi:10.1186/s12863-017-0590-7)
Supplement: Supplementary file 2 — Table S1. List of temperate japonica accessions used. (DOCX 28 kb) [file 12863_2017_590_MOESM2_ESM.docx]

| No. | IRGC Acc. No. | IRRI GID | DESIGNATION | ORIGIN |
| --- | --- | --- | --- | --- |
| 1 | 117518 | 3635167 | KOTOBUKI MOCHI::IRGC 2545-1 | Japan |
| 2 | 117537 | 4282944 | NEP HOA VANG::IRGC 40748-2 | Viet Nam |
| 3 | 117569 | 3421965 | RIKUTO KEMOCHI::IRGC 2719-1 | Japan |
| 4 | 121242 | 3996284 | 7507-137::IRGC 40081-1 | Japan |
| 5 | 121249 | 4253557 | AMARELO::IRGC 9389-1 | Hungary |
| 6 | 121471 | 3634815 | PI 282203::IRGC 16292-1 | Hungary |
| 7 | 121501 | 3412143 | SHINCHIKU IKU 103::IRGC 10430-1 | Chinese Taipei (Taiwan) |
| 8 | 121541 | 3634954 | WIR 1072::IRGC 57496-1 | Belgium |
| 9 | 121816 | 3420813 | S 102/2::GERVEX 1251-C1 | Portugal |
| 10 | 121817 | 3420814 | S 102::GERVEX 1671-C1 | United States |
| 11 | 121818 | 3420815 | SAEDINENIE::GERVEX 1255-C1 | Bulgaria |
| 12 | 121819 | 3420816 | SAFARI::GERVEX 1256-C1 | Portugal |
| 13 | 121820 | 4243712 | SAGRES::GERVEX 1258-C1 | Portugal |
| 14 | 121968 | 3635596 | ARLESIENNE::GERVEX 1530-C1 | France |
| 15 | 121971 | 3475223 | AUGUSTO::GERVEX 1643-C1 | Italy |
| 16 | 121972 | 3475224 | AUZGUSTA::GERVEX 1634-C1 | Hungary |
| 17 | 121996 | 3475501 | CAMPINO::GERVEX 824-C1 | Portugal |
| 18 | 121998 | 3475242 | CAPATAZ::GERVEX 521-C1 | Spain |
| 19 | 122001 | 3475245 | CARRICO::GERVEX 828-C1 | Portugal |
| 20 | 122007 | 3475251 | CHIPKA::GERVEX 837-C1 | Bulgaria |
| 21 | 122013 | 3475257 | CIGALON::GERVEX 1514-C1 | France |
| 22 | 122016 | 3635619 | CLOT::GERVEX 523-C1 | Spain |
| 23 | 122024 | 3475268 | CT 58::GERVEX 1491-C1 | Colombia |
| 24 | 122043 | 3475287 | ESCARLATE::GERVEX 887-C1 | Portugal |
| 25 | 122045 | 3475289 | FAISCA::GERVEX 900-C1 | Portugal |
| 26 | 122049 | 3475503 | FLIPPER::GERVEX 597-C1 | Italy |
| 27 | 122051 | 3475293 | FRANCES::GERVEX 527-C1 | Spain |
| 28 | 122053 | 3475295 | GHIBLI::GERVEX 187-C1 | Italy |
| 29 | 122056 | 3626299 | GIOVANNI MARCHETTI::GERVEX 62-C1 | Italy |
| 30 | 122078 | 3475320 | HARRA::GERVEX 501-C1 | Australia |
| 31 | 122085 | 3475327 | IBO 400::GERVEX 943-C1 | Portugal |
| 32 | 122121 | 3475359 | ITALPATNA 48::GERVEX 60-C1 | Italy |
| 33 | 122126 | 3635044 | JUBILIENI::GERVEX 992-C1 | Bulgaria |
| 34 | 122142 | 3635049 | KULON::GERVEX 1473-C1 | Russian Federation |
| 35 | 122149 | 3475387 | LOMELLINO::GERVEX 83-C1 | Italy |
| 36 | 122151 | 3475389 | LOTO::GERVEX 104-C1 | Italy |
| 37 | 122152 | 3475390 | LUSITO IRRADIADO 859-85-2::GERVEX 1676-C1 | Portugal |
| 38 | 122153 | 3475391 | LUXOR::GERVEX 1662-C1 | Italy |
| 39 | 122162 | 3475398 | MARENY::GERVEX 535-C1 | Spain |
| 40 | 122175 | 3475411 | MUGA::GERVEX 1099-C1 | Portugal |
| 41 | 122183 | 4253558 | OPALE::GERVEX 1667-C1 | Italy |
| 42 | 122187 | 3475423 | OTA::GERVEX 1145-C1 | Portugal |
| 43 | 122202 | 3635371 | PIEMONTE::GERVEX 81-C1 | Italy |
| 44 | 122203 | 3475439 | PLOVDIV 22::GERVEX 1167-C1 | Bulgaria |
| 45 | 122204 | 3475440 | PLOVDIV 24::GERVEX 1168-C1 | Bulgaria |
| 46 | 122207 | 3635205 | POLIZESTI 28::GERVEX 1170-C1 | Bulgaria |
| 47 | 122208 | 4253040 | PRECOZ 2 F A::GERVEX 1182-C1 | Argentina |
| 48 | 122217 | 3475449 | RIBE 253::GERVEX 54-C1 | Italy |
| 49 | 122219 | 3475451 | RODINA::GERVEX 1234-C1 | Bulgaria |
| 50 | 122222 | 3635212 | ROXANI::GERVEX 1686-C1 | Greece |
| 51 | 122223 | 3475455 | RPC 12::GERVEX 1505-C1 | China |
| 52 | 122229 | 3475461 | RUBI::GERVEX 1247-C1 | Portugal |
| 53 | 122230 | 3475462 | RUBINO::GERVEX 80-C1 | Italy |
| 54 | 122233 | 3475465 | SAKHA 102::GERVEX 1687-C1 | Egypt |
| 55 | 122234 | 3475466 | SAKHA 103::GERVEX 1688-C1 | Egypt |
| 56 | 122235 | 4245123 | SALOIO::GERVEX 1259-C1 | Portugal |
| 57 | 122241 | 3475473 | SANGHAI::GERVEX 1264-C1 | China |
| 58 | 122244 | 3475476 | SELN 244 A 6-20::GERVEX 1273-C1 | Australia |
| 59 | 122249 | 3475481 | SETTANTUNO::GERVEX 1279-C1 | Portugal |
| 60 | 122251 | 3475483 | SHSS 53::GERVEX 550-C1 | Spain |
| 61 | 122253 | 3475485 | SMERALDO::GERVEX 138-C1 | Italy |
| 62 | 122256 | 3475488 | SR 113::GERVEX 553-C1 | Spain |
| 63 | 122257 | 3732618 | SUPER::GERVEX 1304-C1 | Portugal |
| 64 | 122259 | 3475491 | T 757::GERVEX 1316-C1 | India |
| 65 | 122265 | 3475517 | THAIPERLA::GERVEX 696-C1 | United States |
| 66 | 122267 | 3475519 | TIMICH 108::GERVEX 1325-C1 | Romania |
| 67 | 122268 | 3475520 | TOPAZIO::GERVEX 1332-C1 | Portugal |
| 68 | 122271 | 3475523 | ULLAL::GERVEX 556-C1 | Spain |
| 69 | 122273 | 3475525 | VALTEJO::GERVEX 1355-C1 | Portugal |
| 70 | 122300 | 3475552 | YRM 6-2::GERVEX 1508-C1 | Australia |
| 71 | 122303 | 3551458 | DELTA::GERVEX 1519-C1 | France |
| 72 | 125610 | 3994793 | FU LI HONG::IRGC 70250-1 | China |
| 73 | 125620 | 3994802 | M 102::IRGC 76307-1 | United States |
| 74 | 125631 | 4253056 | 81 A 32::IRGC 60162-1 | China |
| 75 | 125679 | 3944027 | BETIS::IRGC 74581-1 | Spain |
| 76 | 125698 | 4245125 | CHALBYEO::IRGC 77639-1 | Korea, Republic Of |
| 77 | 125700 | 3944064 | CHEONJUDO::IRGC 77644-1 | Korea, Republic Of |
| 78 | 125701 | 3944066 | CHIANAN 8::IRGC 90-1 | Chinese Taipei (Taiwan) |
| 79 | 125707 | 4245038 | CHUGOKU 68 HEN::IRGC 72514-1 | Japan |
| 80 | 125709 | 3944079 | CHUNG YI::IRGC 1427-1 | China |
| 81 | 125710 | 3994782 | CI 1600::IRGC 16305-1 | United States |
| 82 | 125712 | 3944084 | CI 9498::IRGC 2134-1 | United States |
| 83 | 125717 | 3944091 | DACHEONGBYEO::IRGC 72533-1 | Korea, Republic Of |
| 84 | 125718 | 3994784 | DA DAO TOU::IRGC 59499-1 | China |
| 85 | 125743 | 4244867 | FUKUSHIMA MOCHI (GLUT)::IRGC 19296-1 | Japan |
| 86 | 125752 | 3944144 | GITANO::IRGC 82424-1 | Italy |
| 87 | 125757 | 3994826 | HAN NUO::IRGC 59591-1 | China |
| 88 | 125758 | 3944153 | HAN NUO::IRGC 82350-1 | China |
| 89 | 125761 | 3944157 | HEUKSANJO::IRGC 55536-1 | Korea, Republic Of |
| 90 | 125764 | 4253054 | HOKUSETSU::IRGC 65705-1 | Japan |
| 91 | 125767 | 3944163 | HUK ZO::IRGC 19760-1 | Korea, Republic Of |
| 92 | 125768 | 4244868 | HWANGJO::IRGC 55547-1 | Korea, Republic Of |
| 93 | 125781 | 3944181 | JAPONES BALILLA::IRGC 5785-1 | Uruguay |
| 94 | 125784 | 3944185 | JEUK DO::IRGC 19775-1 | Korea, Republic Of |
| 95 | 125788 | 3944191 | JO SANG DAE YA::IRGC 90852-1 | Korea, Dpr |
| 96 | 125791 | 4244874 | KALIN::IRGC 77312-1 | Bulgaria |
| 97 | 125794 | 3944200 | KANU DAM::IRGC 29755-1 | Cambodia |
| 98 | 125808 | 4070415 | KOPANCSI KEREK::IRGC 9305-1 | Hungary |
| 99 | 125830 | 4244957 | MAEKJO::IRGC 77666-1 | Korea, Republic Of |
| 100 | 125846 | 3993968 | MURASAHITSUTSURI::IRGC 2493-1 | Japan |
| 101 | 125856 | 4253057 | NONG KE::IRGC 59807-1 | China |
| 102 | 125860 | 3944299 | OEIRAS::IRGC 286-1 | Portugal |
| 103 | 125862 | 3944302 | O. SATIVA::IRGC 12876-1 | Spain |
| 104 | 125888 | 3944341 | ROCCA::IRGC 50351-1 | Italy |
| 105 | 125893 | 3944349 | S 201::IRGC 55230-1 | United States |
| 106 | 125898 | 3944356 | SANT ANDREA::IRGC 65732-1 | Italy |
| 107 | 125899 | 4244880 | SENIA::IRGC 74582-1 | Spain |
| 108 | 125908 | 4245209 | SZANISZLO 2::IRGC 9353-1 | Hungary |
| 109 | 125910 | 3944374 | TAICHUNG 179::IRGC 85-1 | Chinese Taipei (Taiwan) |
| 110 | 125911 | 3993976 | TAICHUNG 65::IRGC 79-1 | Chinese Taipei (Taiwan) |
| 111 | 125915 | 3944382 | TAKAO MOCHI::IRGC 2564-1 | Japan |
| 112 | 125919 | 3944388 | TEPUKE::IRGC 12872-1 | New Zealand |
| 113 | 125920 | 3944389 | TEXAS PATNA 49::IRGC 6077-1 | United States |
| 114 | 125934 | 3944408 | WA BANG::IRGC 19880-1 | Korea, Republic Of |
| 115 | 125938 | 4244882 | WIR 1951::IRGC 51643-1 | Georgia |
| 116 | 125939 | 4244883 | WIR 884::IRGC 51591-1 | Norway |
| 117 | 125945 | 4244892 | YONG AN HUK::IRGC 19891-1 | Korea, Republic Of |
| 118 | 125980 | 4244631 | IR 73688-57-2::IRGC 117383-1 | Philippines |
| 119 | 126027 | 4244635 | IR 68333-R-R-B-19::IRGC 117381-1 | Philippines |
| 120 | 126074 | 3944348 | NEP NGAU::IRGC 78369-1 | Viet Nam |
| 121 | 126113 | 3972940 | 68-2::IRGC 14546-1 | France |
| 122 | 126118 | 3972945 | M 203::IRGC 76309-1 | United States |
| 123 | 126120 | 3972947 | NORIN 6::IRGC 2633-1 | Japan |
| 124 | 126185 | 3973326 | BERGREIS::IRGC 3150-1 | Austria |
| 125 | 126198 | 3973339 | DAN YAN NUO::IRGC 4860-1 | China |
| 126 | 126967 | 4244846 | JINBUBYEO::G1 | Korea, Republic Of |
| 127 | 126990 | 4253532 | SUWEON 295::IRGC 58368-1 | Korea, Republic Of |
| 128 | 127036 | 4006019 | BENLLOK::IRGC 3404-1 | Peru |
| 129 | 127058 | 4244884 | M 7::IRGC 34281-1 | United States |
| 130 | 127074 | 4006057 | H 305-84::IRGC 116988-1 | Hungary |
| 131 | 127247 | 4253428 | CANLUBANG::IRGC 69816-1 | Philippines |
| 132 | 127285 | 4070629 | CN 1067::IRGC 65680-1 | United States |
| 133 | 127415 | 4069753 | HONG PI NUO::IRGC 59638-1 | China |
| 134 | 127608 | 4070139 | MAO ZHA NUO::IRGC 70335-1 | China |
| 135 | 127642 | 4070173 | NAGKAYAT::IRGC 584-1 | Philippines |
| 136 | 127798 | 4070305 | SHINCHIKU IKU 97::IRGC 10429-1 | Chinese Taipei (Taiwan) |
| 137 | 127815 | 4070316 | SI WAN 14::IRGC 63019-1 | China |
| 138 | 127834 | 4070334 | TAICHUNG 150::IRGC 80-1 | Chinese Taipei (Taiwan) |
| 139 | 127920 | 4253538 | 4583::IRGC 36894-2 | China |
| 140 | 128194 | 4064141 | TSAO SIAO PEH TAO::IRGC 8265-2 | China |
| 141 | 128209 | 4253169 | 250 KUNGANI 1::IRGC 7370-1 | Japan |
| 142 | 128212 | 4069613 | AIKAWA 44::IRGC 7676-1 | Japan |
| 143 | 128235 | 4253526 | BAI MANG AI ZHONG::IRGC 59408-1 | China |
| 144 | 128240 | 4069641 | BEN KEI::IRGC 7769-1 | Japan |
| 145 | 128252 | 4069653 | C 722323::IRGC 73147-1 | Chinese Taipei (Taiwan) |
| 146 | 128261 | 4069693 | CHINES::IRGC 9316-1 | Unknown |
| 147 | 128262 | 4253536 | CHUBU 17::IRGC 72505-1 | Japan |
| 148 | 128263 | 4253514 | CHUSEI HONEN::IRGC 7777-1 | Japan |
| 149 | 128275 | 4253498 | DECHANGBYEO::IRGC 64858-1 | Korea, Republic Of |
| 150 | 128277 | 4253497 | DEWAMINORI::IRGC 12743-1 | Japan |
| 151 | 128284 | 4069716 | DUAN SHEN ZI::IRGC 73962-1 | China |
| 152 | 128291 | 4069723 | FEI ZHAO 12::IRGC 62683-1 | China |
| 153 | 128299 | 4069731 | GINMASARI::IRGC 242-1 | Japan |
| 154 | 128303 | 4244728 | GONG SHE 9::IRGC 62693-1 | China |
| 155 | 128305 | 4244730 | GYEONGSAN 1::IRGC 79404-1 | Korea, Republic Of |
| 156 | 128307 | 4253616 | HEI TOU HONG::IRGC 59595-1 | China |
| 157 | 128312 | 4244723 | HUA 24::IRGC 82127-1 | China |
| 158 | 128315 | 4069772 | IAS 22-8 PALMAR::IRGC 26058-1 | Brazil |
| 159 | 128326 | 4069783 | K 113::IRGC 34107-1 | India |
| 160 | 128347 | 4069804 | KINUGASAWASE::IRGC 2609-1 | Japan |
| 161 | 128364 | 4069821 | LIGEN 2::IRGC 82398-1 | China |
| 162 | 128365 | 4244932 | LIJIAN 942::IRGC 82399-1 | China |
| 163 | 128367 | 4244922 | LITCHIKIANG::IRGC 7287-1 | China |
| 164 | 128370 | 4253211 | LUAN DAO::IRGC 59762-1 | China |
| 165 | 128384 | 4253197 | MA SHE 8::IRGC 62750-1 | China |
| 166 | 128396 | 4253575 | MUNJI::IRGC 70928-1 | Pakistan |
| 167 | 128409 | 4245032 | NORIN 21::IRGC 493-1 | Japan |
| 168 | 128414 | 4069896 | OITA MII 120::IRGC 7696-1 | Japan |
| 169 | 128434 | 4069942 | PL 3165::IRGC 62827-1 | China |
| 170 | 128445 | 4069953 | RAI MANULA::IRGC 64138-1 | Nepal |
| 171 | 128455 | 4069963 | SACHIKAZE::IRGC 10891-1 | Japan |
| 172 | 128469 | 4253212 | SHA TIAO TSAO::IRGC 7339-1 | Unknown |
| 173 | 128499 | 4253489 | WAN GENG BAI DAO TOU::IRGC 59948-1 | China |
| 174 | 128501 | 4244929 | WEONJU 8::IRGC 90845-1 | Korea, Republic Of |
| 175 | 128502 | 4244930 | WIR 2091::IRGC 57536-1 | Georgia |
| 176 | 128505 | 4253206 | XINTUAN HEI GU::IRGC 56159-1 | China |
| 177 | 128507 | 4244740 | YE ZO::IRGC 19888-1 | Korea, Republic Of |
| 178 | 128508 | 4070041 | YI SUI QI::IRGC 60088-1 | China |
| 179 | 128509 | 4070042 | YUNLEN 13::IRGC 82402-1 | China |
| 180 | 128514 | 4070047 | ZUIHOU::IRGC 66982-1 | Japan |
| 181 | 128517 | 4070050 | CT 45::IRGC 34027-2 | India |
| 182 | 128519 | 4070052 | IREQUIN::IRGC 44476-2 | Philippines |
| 183 | 131960 | 4438291 | 91-382::IRGC 63464-1 | Bhutan |
| 184 | 131998 | 4438329 | KARA SHALI::IRGC 9293-1 | Unknown |
| 185 | 132020 | 4438351 | NOINJO::IRGC 77669-1 | Korea, Republic Of |
| 186 | 132043 | 4438374 | TAICHUNG 188::IRGC 78209-1 | Chinese Taipei (Taiwan) |
| 187 | 132271 | 4438602 | 7516-14::IRGC 40090-3 | Japan |
| 188 | 132313 | 4438644 | 36037-1::IRGC 60177-2 | China |
| 189 | 132323 | 4438654 | BACK KYUNG ZO::IRGC 19698-2 | Korea, Republic Of |
| 190 | 132370 | 4438701 | SSAL BYEO::IRGC 19867-2 | Korea, Republic Of |
| 191 | 132425 | 4454076 | K 78-13::IRGC 36794-2 | India |
