# Supplementary material for: Genome-wide association study of seedling stage salinity tolerance in temperate japonica rice germplasm
Source: BMC Genet. 2018 Jan 3;19:2. doi: 10.1186/s12863-017-0590-7 (PMC5753436; doi:10.1186/s12863-017-0590-7)
Supplement: Supplementary file 3 — Table S2. Standard evaluation score (SES) of visual salt injury at seedling stage. (DOCX 12 kb) [file 12863_2017_590_MOESM3_ESM.docx]

| Score | Observation | Tolerance |
| --- | --- | --- |
| 1 | Normal growth, no leaf symptoms | Highly tolerant |
| 3 | Nearly normal growth, but leaf tips or few leaves whitish and rolled | Tolerant |
| 5 | Growth severely retarded; most leaves rolled; only a few are elongating | Moderately tolerant |
| 7 | Complete cessation of growth; most leaves dry; some plants dying | Susceptible |
| 9 | Almost all plants dead or dying | Highly susceptible |
