# Supplementary material for: Genome-wide association study of seedling stage salinity tolerance in temperate japonica rice germplasm
Source: BMC Genet. 2018 Jan 3;19:2. doi: 10.1186/s12863-017-0590-7 (PMC5753436; doi:10.1186/s12863-017-0590-7)
Supplement: Supplementary file 4 — Table S3. Statistics of the measured traits. (DOCX 14 kb) [file 12863_2017_590_MOESM4_ESM.docx]

| Variable | Mean | SE | StDev | Minimum | Maximum |
| --- | --- | --- | --- | --- | --- |
| SES12 | 6.49 | 0.11 | 1.51 | 3.00 | 9.00 |
| SES18 | 8.00 | 0.10 | 1.33 | 4.33 | 9.00 |
| PD12 | 66.73 | 1.57 | 20.89 | 15.00 | 100.00 |
| PD18 | 86.34 | 1.37 | 18.17 | 33.33 | 100.00 |
| SDW0 | 0.21 | 0.01 | 0.07 | 0.07 | 0.46 |
| SDW12 | 0.08 | 0.00 | 0.03 | 0.03 | 0.23 |
| SDWD | 0.57 | 0.01 | 0.18 | 0.02 | 0.88 |
| SNa0 | 0.50 | 0.02 | 0.27 | 0.17 | 1.65 |
| SNa12 | 2.61 | 0.09 | 1.14 | 0.67 | 5.73 |
| SNaD | -5.38 | 0.31 | 4.16 | -23.00 | -0.28 |
| SK0 | 1.50 | 0.06 | 0.75 | 0.10 | 3.67 |
| SK12 | 1.36 | 0.04 | 0.48 | 0.37 | 2.63 |
| SKD | -0.26 | 0.09 | 1.18 | -9.33 | 0.87 |
| Na/K0 | 0.84 | 0.05 | 0.70 | 0.10 | 3.57 |
| Na/K12 | 4.85 | 0.31 | 4.13 | 0.99 | 20.75 |
| Na/KD | -9.29 | 1.00 | 13.27 | -102.05 | 0.60 |
