# Supplementary material for: Genome-wide association study of seedling stage salinity tolerance in temperate japonica rice germplasm
Source: BMC Genet. 2018 Jan 3;19:2. doi: 10.1186/s12863-017-0590-7 (PMC5753436; doi:10.1186/s12863-017-0590-7)
Supplement: Supplementary file 7 — Table S4. List of salinity tolerant accessions at seedling stage based on SES12. (DOCX 16 kb) [file 12863_2017_590_MOESM7_ESM.docx]

| Number | SEQID | IRGC Acc. No. | GID | Variety Name | ORIGIN | SES12 | SES18 |
| --- | --- | --- | --- | --- | --- | --- | --- |
| 1 | IRIS_313-10437 | 128409 | 4245032 | NORIN 21 | Japan | 4.3 | 4.3 |
| 2 | IRIS_313-9140 | 125631 | 4253056 | 81 A 32 | China | 5.0 | 4.3 |
| 3 | IRIS_313-11586 | 132313 | 4438644 | 36037_1 | China | 5.0 | 4.3 |
| 4 | IRIS_313-8033 | 122149 | 3475387 | LOMELLINO | Italy | 4.0 | 4.3 |
| 5 | IRIS_313-10631 | 127798 | 4070305 | SHINCHIKU IKU 97 | Taiwan | 3.0 | 4.7 |
| 6 | IRIS_313-11571 | 128235 | 4253526 | BAI MANG AI ZHONG | China | 3.0 | 5.0 |
| 7 | IRIS_313-8399 | 126113 | 3972940 | 68_2 | France | 3.7 | 5.0 |
| 8 | IRIS_313-8755 | 126120 | 3972947 | NORIN 6 | Japan | 4.0 | 5.7 |
| 9 | IRIS_313-8138 | 122235 | 4245123 | SALOIO | Portugal | 4.3 | 5.7 |
| 10 | IRIS_313-11652 | 128303 | 4244728 | GONG SHE 9 | China | 4.3 | 5.7 |
| 11 | IRIS_313-11981 | 132043 | 4438374 | TAICHUNG 188 | Taiwan | 4.3 | 5.7 |
| 12 | IRIS_313-8151 | 122273 | 3475525 | VALTEJO | Portugal | 4.3 | 6.3 |
| 13 | IRIS_313-10642 | 128455 | 4069963 | SACHIKAZE | Japan | 4.3 | 6.3 |
| 14 | IRIS_313-8735 | 126074 | 3944348 | NEP NGAU | Viet Nam | 3.0 | 7.0 |
| 15 | IRIS_313-8112 | 122007 | 3475251 | CHIPKA | Bulgaria | 4.0 | 7.0 |
| 16 | IRIS_313-15904 | 126967 | 4244846 | JINBUBYEO | Korea Rep. | 4.3 | 7.0 |
| 17 | IRIS_313-11651 | 128291 | 4069723 | FEI ZHAO 12 | China | 4.3 | 7.0 |
| 18 | IRIS_313-10583 | 128194 | 4064141 | TSAO SIAO PEH TAO | China | 3.7 | 7.7 |
| 19 | IRIS_313-10618 | 128261 | 4069693 | CHINES | Unknown | 3.7 | 7.7 |
| 20 | IRIS_313-8502 | 125620 | 3994802 | M 102 | United States | 4.3 | 7.7 |
| 21 | IRIS_313-8216 | 122222 | 3635212 | ROXANI | Greece | 4.3 | 7.7 |
| 22 | IRIS_313-8136 | 121819 | 3420816 | SAFARI | Portugal | 4.3 | 7.7 |
| 23 | IRIS_313-10429 | 127834 | 4070334 | TAICHUNG 150 | Taiwan | 4.3 | 7.7 |
| 24 | IRIS_313-10057 | 125707 | 4245038 | CHUGOKU 68 HEN | Japan | 3.7 | 9.0 |
| 25 | IRIS_313-10059 | 125717 | 3944091 | DACHEONGBYEO | Korea Rep. | 4.3 | 9.0 |
| 26 | IRIS_313-8127 | 122207 | 3635205 | POLIZESTI 28 | Bulgaria | 4.3 | 9.0 |
| 27 | IRIS_313-8165 | 122013 | 3475257 | CIGALON | France | 4.3 | 9.0 |
