# Supplementary material for: Genome-wide association study of seedling stage salinity tolerance in temperate japonica rice germplasm
Source: BMC Genet. 2018 Jan 3;19:2. doi: 10.1186/s12863-017-0590-7 (PMC5753436; doi:10.1186/s12863-017-0590-7)
Supplement: Supplementary file 10 — Table S5. Hyplotype of SKC1 gene (Oso1g20160, OsHKT1 Na+ transporter) in salinity-tolerant varieties (Nona Bokra and Pokkali) and 191 temperate japonica accessions. (DOCX 35 kb) [file 12863_2017_590_MOESM10_ESM.docx]

| chrom | 1 | 1 | 1 | 1 | 1 | 1 |
| --- | --- | --- | --- | --- | --- | --- |
| pos | 11458379 | 11460129 | 11462157 | 11462282 | 11462725 | 11462894 |
| SNP | 10111458379 | 10111460129 | 10111462157 | 10111462282 | 10111462725 | 10111462894 |
| Varieties/alleles | T/C | G/C | G/C | G/C | C/T | C/T |
| NONA BOKRA | C | C | G | C | T | C |
| POKKALI | C | C | G | C | C | T |
| SANT ANDREA::IRGC 65732-1 | T | G | G | G | C | C |
| DACHEONGBYEO::IRGC 72533-1 | T | G | G | G | C | C |
| BETIS::IRGC 74581-1 | C | N | G | G | C | C |
| SENIA::IRGC 74582-1 | T | G | G | G | C | C |
| KALIN::IRGC 77312-1 | C | G | G | G | C | C |
| CHALBYEO::IRGC 77639-1 | T | G | G | G | C | C |
| CHEONJUDO::IRGC 77644-1 | C | G | C | G | C | C |
| MAEKJO::IRGC 77666-1 | T | G | N | G | C | C |
| GITANO::IRGC 82424-1 | C | G | G | G | C | C |
| HAN NUO::IRGC 59591-1 | T | G | G | G | C | C |
| HAN NUO::IRGC 82350-1 | C | G | G | G | C | T |
| H 305-84::IRGC 116988-1 | T | G | G | G | C | C |
| IR 68333-R-R-B-19::IRGC 117381-1 | T | G | G | G | C | C |
| IR 73688-57-2::IRGC 117383-1 | T | G | G | G | C | C |
| TAICHUNG 150::IRGC 80-1 | C | G | C | G | C | C |
| GINMASARI::IRGC 242-1 | T | G | G | G | C | C |
| NORIN 21::IRGC 493-1 | T | G | G | G | C | C |
| NAGKAYAT::IRGC 584-1 | C | G | G | G | C | C |
| KINUGASAWASE::IRGC 2609-1 | T | G | G | G | C | C |
| LITCHIKIANG::IRGC 7287-1 | C | G | G | G | C | C |
| SHA TIAO TSAO::IRGC 7339-1 | T | G | G | G | C | C |
| 250 KUNGANI 1::IRGC 7370-1 | C | G | C | G | C | C |
| AIKAWA 44::IRGC 7676-1 | T | G | G | G | C | C |
| OITA MII 120::IRGC 7696-1 | T | G | G | G | C | C |
| BEN KEI::IRGC 7769-1 | T | G | G | G | C | C |
| CHUSEI HONEN::IRGC 7777-1 | T | G | G | G | C | C |
| TSAO SIAO PEH TAO::IRGC 8265-2 | C | N | G | G | C | C |
| CHINES::IRGC 9316-1 | T | G | G | G | C | C |
| SHINCHIKU IKU 97::IRGC 10429-1 | C | G | C | G | C | C |
| SACHIKAZE::IRGC 10891-1 | T | G | G | G | C | C |
| DEWAMINORI::IRGC 12743-1 | T | G | G | G | C | C |
| BACK KYUNG ZO::IRGC 19698-2 | T | G | G | G | C | C |
| SSAL BYEO::IRGC 19867-2 | T | G | G | G | C | C |
| YE ZO::IRGC 19888-1 | T | G | G | G | C | C |
| IAS 22-8 PALMAR::IRGC 26058-1 | T | G | G | G | C | C |
| 4583::IRGC 36894-2 | T | G | G | G | C | C |
| XINTUAN HEI GU::IRGC 56159-1 | C | G | G | G | C | C |
| WIR 2091::IRGC 57536-1 | T | G | G | G | C | C |
| BAI MANG AI ZHONG::IRGC 59408-1 | C | G | G | G | C | C |
| HEI TOU HONG::IRGC 59595-1 | C | C | N | G | C | C |
| HONG PI NUO::IRGC 59638-1 | C | G | G | G | C | C |
| LUAN DAO::IRGC 59762-1 | C | N | G | G | C | C |
| WAN GENG BAI DAO TOU::IRGC 59948-1 | T | G | G | G | C | C |
| YI SUI QI::IRGC 60088-1 | C | G | G | G | C | C |
| 36037-1::IRGC 60177-2 | T | G | G | G | C | C |
| GONG SHE 9::IRGC 62693-1 | C | G | G | G | C | C |
| MA SHE 8::IRGC 62750-1 | C | G | G | G | C | C |
| PL 3165::IRGC 62827-1 | T | G | G | G | C | C |
| SI WAN 14::IRGC 63019-1 | C | G | G | G | C | C |
| 91-382::IRGC 63464-1 | T | G | G | G | C | C |
| RAI MANULA::IRGC 64138-1 | C | G | C | G | C | C |
| DECHANGBYEO::IRGC 64858-1 | T | G | G | G | C | C |
| CN 1067::IRGC 65680-1 | T | G | G | G | C | C |
| ZUIHOU::IRGC 66982-1 | T | G | G | G | C | C |
| CANLUBANG::IRGC 69816-1 | C | G | G | G | C | C |
| MAO ZHA NUO::IRGC 70335-1 | T | G | G | G | C | C |
| MUNJI::IRGC 70928-1 | T | G | G | G | C | C |
| CHUBU 17::IRGC 72505-1 | C | G | C | G | C | C |
| C 722323::IRGC 73147-1 | T | G | N | G | C | C |
| DUAN SHEN ZI::IRGC 73962-1 | C | G | G | G | C | C |
| NOINJO::IRGC 77669-1 | T | G | G | G | C | C |
| TAICHUNG 188::IRGC 78209-1 | C | N | C | G | C | C |
| GYEONGSAN 1::IRGC 79404-1 | T | G | G | G | C | C |
| HUA 24::IRGC 82127-1 | T | G | G | G | C | C |
| LIGEN 2::IRGC 82398-1 | C | G | G | G | C | C |
| LIJIAN 942::IRGC 82399-1 | C | G | G | G | C | C |
| YUNLEN 13::IRGC 82402-1 | C | G | G | G | C | C |
| WEONJU 8::IRGC 90845-1 | T | G | G | G | C | C |
| JINBUBYEO::G1 | T | G | G | G | C | C |
| RIBE 253::GERVEX 54-C1 | T | G | G | G | C | C |
| ITALPATNA 48::GERVEX 60-C1 | T | G | G | G | C | C |
| GIOVANNI MARCHETTI::GERVEX 62-C1 | T | G | G | G | C | C |
| RUBINO::GERVEX 80-C1 | T | G | G | G | C | C |
| LOMELLINO::GERVEX 83-C1 | T | G | G | G | C | C |
| LOTO::GERVEX 104-C1 | T | G | G | G | C | C |
| SMERALDO::GERVEX 138-C1 | T | G | G | G | C | C |
| GHIBLI::GERVEX 187-C1 | T | G | G | G | C | C |
| HARRA::GERVEX 501-C1 | T | G | G | G | C | C |
| CAPATAZ::GERVEX 521-C1 | C | G | G | G | C | C |
| CLOT::GERVEX 523-C1 | T | G | G | G | C | C |
| FRANCES::GERVEX 527-C1 | T | G | G | G | C | C |
| MARENY::GERVEX 535-C1 | T | G | G | G | C | C |
| SHSS 53::GERVEX 550-C1 | T | G | G | G | C | C |
| SR 113::GERVEX 553-C1 | T | G | G | G | C | C |
| ULLAL::GERVEX 556-C1 | T | G | G | G | C | C |
| FLIPPER::GERVEX 597-C1 | T | G | G | G | C | C |
| THAIPERLA::GERVEX 696-C1 | T | G | G | G | C | C |
| CAMPINO::GERVEX 824-C1 | T | N | G | G | C | C |
| CARRICO::GERVEX 828-C1 | T | G | G | G | C | C |
| CHIPKA::GERVEX 837-C1 | T | G | G | G | C | C |
| ESCARLATE::GERVEX 887-C1 | T | G | G | G | C | C |
| FAISCA::GERVEX 900-C1 | T | G | G | G | C | C |
| IBO 400::GERVEX 943-C1 | C | G | G | G | C | C |
| JUBILIENI::GERVEX 992-C1 | T | G | G | G | C | C |
| MUGA::GERVEX 1099-C1 | T | G | G | G | C | C |
| OTA::GERVEX 1145-C1 | T | G | G | G | C | C |
| PLOVDIV 22::GERVEX 1167-C1 | T | G | G | G | C | C |
| PLOVDIV 24::GERVEX 1168-C1 | T | G | G | G | C | C |
| PRECOZ 2 F A::GERVEX 1182-C1 | T | G | G | G | C | C |
| RODINA::GERVEX 1234-C1 | T | G | G | G | C | C |
| RUBI::GERVEX 1247-C1 | T | G | G | G | C | C |
| S 102/2::GERVEX 1251-C1 | T | G | G | G | C | C |
| SAEDINENIE::GERVEX 1255-C1 | T | G | G | G | C | C |
| SAFARI::GERVEX 1256-C1 | T | G | G | G | C | C |
| SALOIO::GERVEX 1259-C1 | T | G | G | G | C | C |
| SANGHAI::GERVEX 1264-C1 | T | G | G | G | C | C |
| SELN 244 A 6-20::GERVEX 1273-C1 | C | G | G | G | C | C |
| SETTANTUNO::GERVEX 1279-C1 | T | G | G | G | C | C |
| SUPER::GERVEX 1304-C1 | C | G | G | G | C | C |
| T 757::GERVEX 1316-C1 | T | G | G | G | C | C |
| TIMICH 108::GERVEX 1325-C1 | T | G | G | G | C | C |
| TOPAZIO::GERVEX 1332-C1 | T | G | G | G | C | C |
| VALTEJO::GERVEX 1355-C1 | C | G | G | G | C | C |
| KULON::GERVEX 1473-C1 | T | G | G | G | C | C |
| RPC 12::GERVEX 1505-C1 | T | G | G | G | C | C |
| YRM 6-2::GERVEX 1508-C1 | T | G | G | G | C | C |
| CIGALON::GERVEX 1514-C1 | T | G | G | G | C | C |
| DELTA::GERVEX 1519-C1 | T | G | G | G | C | C |
| ARLESIENNE::GERVEX 1530-C1 | C | G | G | G | C | C |
| AUZGUSTA::GERVEX 1634-C1 | T | G | G | G | C | C |
| AUGUSTO::GERVEX 1643-C1 | C | G | G | G | C | C |
| LUXOR::GERVEX 1662-C1 | T | G | G | G | C | C |
| S 102::GERVEX 1671-C1 | T | G | G | G | C | C |
| LUSITO IRRADIADO 859-85-2::GERVEX 1676-C1 | T | G | G | G | C | C |
| ROXANI::GERVEX 1686-C1 | T | G | G | G | C | C |
| SAKHA 102::GERVEX 1687-C1 | T | N | G | G | C | C |
| SAKHA 103::GERVEX 1688-C1 | T | G | N | G | C | C |
| FU LI HONG::IRGC 70250-1 | N | G | G | G | C | C |
| 68-2::IRGC 14546-1 | T | N | G | G | C | C |
| M 203::IRGC 76309-1 | T | G | G | G | C | C |
| DAN YAN NUO::IRGC 4860-1 | T | G | G | G | C | C |
| M 102::IRGC 76307-1 | T | G | G | G | C | C |
| M 7::IRGC 34281-1 | T | G | G | G | C | C |
| NEP HOA VANG::IRGC 40748-2 | C | G | G | G | C | C |
| NEP NGAU::IRGC 78369-1 | C | G | G | G | C | C |
| NORIN 6::IRGC 2633-1 | T | G | G | G | C | C |
| S 201::IRGC 55230-1 | T | G | G | G | C | C |
| WIR 1072::IRGC 57496-1 | T | N | N | G | C | C |
| KOTOBUKI MOCHI::IRGC 2545-1 | T | N | G | G | C | C |
| TAKAO MOCHI::IRGC 2564-1 | T | G | G | G | C | C |
| 81 A 32::IRGC 60162-1 | T | G | G | G | C | C |
| HOKUSETSU::IRGC 65705-1 | T | G | G | G | C | C |
| KANU DAM::IRGC 29755-1 | T | G | G | G | C | C |
| SHINCHIKU IKU 103::IRGC 10430-1 | N | G | C | G | C | C |
| HWANGJO::IRGC 55547-1 | T | G | G | G | C | C |
| CI 9498::IRGC 2134-1 | T | G | G | G | C | C |
| 7507-137::IRGC 40081-1 | C | G | C | G | C | C |
| CI 1600::IRGC 16305-1 | T | G | N | G | C | C |
| NONG KE::IRGC 59807-1 | C | G | N | G | C | C |
| RIKUTO KEMOCHI::IRGC 2719-1 | C | G | G | G | C | C |
| TEXAS PATNA 49::IRGC 6077-1 | T | G | G | G | C | C |
| JO SANG DAE YA::IRGC 90852-1 | T | G | G | G | C | C |
| TAICHUNG 65::IRGC 79-1 | C | G | C | G | C | C |
| TAICHUNG 179::IRGC 85-1 | C | G | C | G | C | C |
| CHIANAN 8::IRGC 90-1 | C | G | C | G | C | C |
| DA DAO TOU::IRGC 59499-1 | T | G | G | G | C | C |
| OEIRAS::IRGC 286-1 | T | G | G | G | C | C |
| MURASAHITSUTSURI::IRGC 2493-1 | C | G | C | G | C | C |
| BERGREIS::IRGC 3150-1 | C | G | C | G | C | C |
| BENLLOK::IRGC 3404-1 | T | G | G | G | C | C |
| KOPANCSI KEREK::IRGC 9305-1 | T | G | G | N | C | C |
| SZANISZLO 2::IRGC 9353-1 | T | G | G | G | C | C |
| AMARELO::IRGC 9389-1 | N | N | N | G | C | C |
| TEPUKE::IRGC 12872-1 | T | G | G | G | C | C |
| O. SATIVA::IRGC 12876-1 | T | G | G | G | C | C |
| PI 282203::IRGC 16292-1 | T | G | N | G | C | C |
| FUKUSHIMA MOCHI (GLUT)::IRGC 19296-1 | C | G | C | G | C | C |
| HUK ZO::IRGC 19760-1 | T | G | G | G | C | C |
| JEUK DO::IRGC 19775-1 | T | G | G | G | C | C |
| WA BANG::IRGC 19880-1 | T | G | G | G | C | C |
| YONG AN HUK::IRGC 19891-1 | C | G | C | G | C | C |
| ROCCA::IRGC 50351-1 | T | G | G | G | C | C |
| WIR 884::IRGC 51591-1 | T | G | G | G | C | C |
| WIR 1951::IRGC 51643-1 | T | G | G | G | C | C |
| HEUKSANJO::IRGC 55536-1 | T | G | G | G | C | C |
| SUWEON 295::IRGC 58368-1 | T | G | G | G | C | C |
